# Partial least squares multimodal analysis of brain network correlates of language deficits in aphasia

**DOI:** 10.1093/braincomms/fcaf246

**Published:** 2025-06-19

**Authors:** Sigfus Kristinsson, Dirk B den Ouden, Christopher Rorden, Roger Newman-Norlund, Lisa Johnson, Janina Wilmskoetter, Ezequiel Gleichgerrcht, Argye E Hillis, Gregory Hickok, Julius Fridriksson, Leonardo Bonilha

**Affiliations:** Department of Communication Sciences and Disorders, University of South Carolina, Columbia, SC 29208, USA; Department of Communication Sciences and Disorders, University of South Carolina, Columbia, SC 29208, USA; Department of Psychology, University of South Carolina, Columbia, SC 29208, USA; Department of Psychology, University of South Carolina, Columbia, SC 29208, USA; Department of Communication Sciences and Disorders, University of South Carolina, Columbia, SC 29208, USA; College of Health Professions, Medical University of South Carolina, Charleston, SC 29425, USA; Department of Neurology, Emory University School of Medicine, Atlanta, GA 30322, USA; Department of Neurology, Johns Hopkins School of Medicine, Baltimore, MD 21287, USA; Department of Cognitive Sciences, School of Social Sciences, University of California, Irvine, CA 92697, USA; Department of Communication Sciences and Disorders, University of South Carolina, Columbia, SC 29208, USA; Department of Neurology, University of South Carolina School of Medicine, Columbia, SC 29203, USA

**Keywords:** aphasia, neuroimaging, partial least squares

## Abstract

Lesion-symptom mapping techniques are essential to determine brain regions critical for language functions. However, high collinearity in neuroimaging and behavioural data remains a challenge for distinguishing neural substrates supporting multiple language domains (shared variance) and those subserving specific language functions (unique variance). Here, we employed a novel approach to multimodal lesion-symptom mapping using multivariate partial least squares regression to delineate the latent structure of lesion-behavioural mapping in aphasia and decompose the shared and unique neural determinants of language impairments. A total of 86 participants with chronic (>12-month post-stroke) aphasia after left hemisphere strokes were examined. Language impairment was assessed with the Western Aphasia Battery-Revised, and brain damage was defined by multimodal neuroimaging (including lesion characteristics, structural and functional connectivity, volumetric measures and functional activity). Neuroimaging modality-specific models were constructed to evaluate the shared versus unique lesion anatomy associated with performance across Western Aphasia Battery-Revised subtests: auditory comprehension, naming, repetition and spontaneous speech. Model accuracy was validated using leave-one-out cross-validation. Latent decomposition revealed that 50% of the covariance between neuroimaging data and language performance was explained by two to six latent variables across models. The spontaneous speech subtest emerged as the most influential language measure across all models, with damage to regions surrounding the perisylvian fissure accounting for the largest amount of shared variance across Western Aphasia Battery-Revised subtests. Critically, the highest-ranking features represented in the latent variable models yielded moderately accurate simultaneous prediction for all language measures (highest *r*: auditory comprehension = 0.45; naming = 0.39; repetition = 0.38; spontaneous speech = 0.42), suggesting that clinically salient language impairments largely reflect damage to shared anatomical networks. Projection of subtest scores onto latent variables revealed that integrity of distributed left and right cortical and subcortical regions uniquely accounted for 5.0–27.9% of residual variance across subtests, with auditory comprehension involving the most extensive network of unique brain regions. These results highlight that dissociating shared versus unique lesion-symptom associations is important for understanding the neural basis of aphasia. Shared lesion anatomy involving perisylvian regions broadly impacts multiple language domains, while distributed regions uniquely explain deficits in specific language domains (e.g. auditory comprehension). These insights improve our understanding of post-stroke aphasia and facilitate future development of more precise, personalized treatment strategies based on each individual’s neuroanatomy.

## Teaser

Kristinsson *et al*. combined multimodal neuroimaging and partial least squares regression to examine the lesion anatomy in post-stroke aphasia, concluding that damage to areas surrounding the perisylvian fissure negatively affects performance across multiple language domains, whereas damage to distributed cortical and subcortical networks was uniquely associated with language domain-specific impairment.

## Graphical Abstract

**Graphical Abstract:**
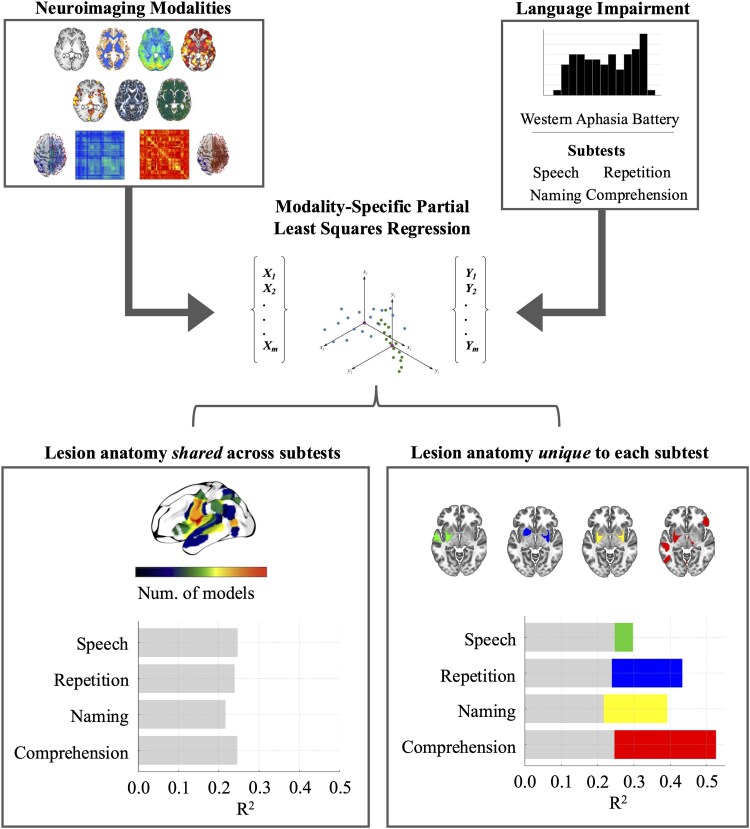

## Introduction

Language is a complex higher-order cognitive function^1,2^ that relies on distributed brain networks in addition to localized regions that subserve specific linguistic processes.^3-7^ A lesion affecting language-specific regions commonly results in aphasia—a debilitating condition defined by the inability to produce or comprehend language.^8^ The most common cause of aphasia is stroke, and understanding the relationship between stroke lesion anatomy and language impairment has historically been a cornerstone of cognitive neuroscience. From early necropsy studies by Pierre Paul Broca or Karl Wernicke to more recent computational neuroimaging approaches, lesion-behavioural correlates provide crucial causal evidence for the critical role of specific brain regions in linguistic tasks, as opposed to non-critical task associations.

The relationship between lesion anatomy and language deficits in aphasia has been extensively studied, with numerous investigations building on the classical lesion-symptom mapping (LSM) framework introduced by Bates *et al*. in 2003.^9-27^ However, one of the main challenges in LSM, particularly in language research, is distinguishing how interrelated behavioural measures correspond to common brain networks as opposed to how they dissociate into distinct brain regions. Behavioural variables frequently exhibit covariance since they often reflect overlapping cognitive domains, and post-stroke brain damage typically involves adjacent regions within the perfusion territories of commonly affected arteries.^28,29^ This makes it difficult to separate *shared* versus *unique* brain–behaviour relationships—where ‘shared’ refers to behaviourally interrelated components mapping to connected brain networks and ‘unique’ refers to localized anatomical–behavioural associations.

This challenge is particularly vexing in aphasia since commonly used clinical assessments include multiple measures that share considerable covariance. Moreover, strokes resulting in aphasia typically involve the middle cerebral artery (MCA) territory, and brain regions within the MCA perfusion territory are rarely damaged in isolation.^28,30^ For instance, in proximal occlusions of the anterior branch of the MCA, the anterior insula is commonly damaged along with the inferior frontal gyrus. Given these complexities, novel approaches are needed to better understand the underlying structure of multivariate behavioural *and* neuroimaging data as they project onto each other in lesion studies of language.

To address these limitations, we applied partial least squares regression (PLSR)—a powerful multivariate approach that offers unique advantages in LSM by quantifying the interdependence between brain–behaviour associations.^31,32^ Unlike traditional mass univariate methods, which are limited in their ability to account for the covariance between behavioural measures and anatomical regions, PLSR is particularly effective in identifying latent components that explain the covariance between neuroimaging data and behavioural outcomes.^33^ Moreover, PLSR involves dimensionality reduction while maintaining interpretability, which is a key advantage when dealing with high-dimensional neuroimaging and behavioural data sets.

In contrast to multivariate approaches leveraged in prior research in aphasia,^21,24,34,35^ including canonical correlation analysis (CCA)^26,36-38^ or principal component analysis (PCA),^19,39,40^ PLSR offers more tailored dimensionality reduction that directly reflects the covariance structure between predictors (e.g. neuroimaging data) and responses (e.g. language scores). Unlike PCA, which focuses solely on maximizing variance within each data set separately, or CCA, which seeks maximal correlations between data sets without considering shared variance in a targeted manner, PLSR optimizes for the projection that best explains the covariance between the data sets.^31^ This allows PLSR to more effectively capture complex brain–behaviour interactions that underlie language deficits in aphasia. By extension, PLSR is particularly well suited for studying aphasia from a network perspective, enabling more precise prediction of language outcomes by accounting for complex interactions across brain regions. This approach not only refines prognostic accuracy but also supports the development of targeted, individualized treatment strategies tailored to each patient’s unique neuroanatomical profile.

Specifically, in an effort to characterize the underlying relationship between lesion damage and language impairment in chronic post-stroke aphasia, we combined PLSR and multiple facets of brain damage as defined by neuroimaging, namely, (i) lesion characteristics, including manually delineated and automatic probabilistic (i3mT1) LSM derived from T1- and T2-weighted imaging; (ii) connectomics (functional and structural connectome optimized for post-stroke connectome LSM) from high-resolution diffusion tensor imaging tractography and resting-state functional MRI; (iii) grey matter (GM) and white matter (WM) tissue integrity [voxel-based morphometry (VBM)] from T1-weighted images; (iv) task-based fMRI; (v) tissue microstructure derived from diffusion MRI; and (vi) tissue perfusion (cerebral blood flow) measured with arterial spin labelling (ASL).

To determine the primary anatomical factors associated with language deficits across these modalities, we applied singular value decomposition (SVD) within the PLSR framework to extract latent variables representing patterns of lesion associated with scores on the four subtests of the Western Aphasia Battery-Revised (WAB-R)^41^: spontaneous speech, naming, repetition and auditory comprehension. The WAB-R was chosen due to its clinical utility, its widely adopted use in language research and the inherent covariance across subtests.^42^ Thus, the identification of shared and unique lesion determinants could be impactful to aid its use and interpretation in both clinical and research perspectives.

Finally, leave-one-out cross-validated machine learning models were constructed to evaluate (i) the *shared* contributions of both neuroimaging and behavioural factors, identifying lesion anatomy associated with global impairment across all WAB-R subtests, and (ii) the *unique* lesion anatomy related to each subtest score. The primary results are presented in terms of variable importance in projection (VIP) scores, which quantify the most influential brain regions across latent variables in each neuroimaging modality-specific PLSR model (i.e. reflecting shared anatomical substrates), and beta coefficients derived by projecting WAB-R subtest scores onto the latent brain–behaviour components (i.e. reflecting unique anatomical substrates). Moreover, the multimodal neuroimaging approach offers multiple complimentary perspectives, providing a comprehensive evaluation of the neurobiological basis of chronic post-stroke aphasia.

## Methods

### Study design and participants

We retrospectively evaluated an existing data set from 107 participants with a history of one or more chronic (at least 12 months prior to enrolment) left hemisphere strokes. The participants evaluated in this study were part of the POLAR (*P*redicting *O*utcomes of *La*nguage *R*ehabilitation in Aphasia) trial (clinicaltrials.gov identifier: NCT03416738; for details on the study protocol, see Kristinsson *et al*.^43^). As part of the POLAR study protocol, all participants underwent thorough behavioural testing and a neuroimaging workup. All participants were recruited through local advertisement at the University of South Carolina (Columbia, SC) or at the Medical University of South Carolina (Charleston, SC). The study was approved by the Institutional Review Boards of both institutions.

From the data set of 107 participants, those without aphasia, i.e. with a WAB-R Aphasia Quotient (AQ) > 93.8, or those without all neuroimaging modalities were excluded from this study. Therefore, the resulting cohort was composed of 86 participants and subsequent analyses refer to these 86 participants. Characteristics of the study sample are presented in Table 1.

**Table 1:** Participants’ demographic characteristics

Variable (*N* = 86)	Mean (SD)/count
Sex, F/M	34/52
Handedness, R/L/A	76/9/1
Stroke type, I/H/other	57/21/8
Race, W/AA/Asian	64/21/1
Lesion volume (cc)	125.7 (93.0)
Test age, y	60.6 (11.2)
Stroke age, y	56.5 (11.7)
Months post-onset	47.8 (48.9)
Education, y	15.5 (2.3)
NIHSS	6.2 (3.9)
ASRS AOS	1.7 (1.6)
WAIS-IV Matrix Reasoning task	11.9 (5.7)
WAB-AQ	59.4 (23.0)
Aphasia type	
Anomic	25
Broca’s	40
Conduction	12
Global	4
Trans-cortical motor	1
Wernicke’s	4

A, ambidextrous; AA, African American; AOS, Apraxia of Speech; ASRS, Apraxia of Speech Rating Scale; F, female; H, haemorrhagic; I, ischaemic; L, left; M, male; NIHSS, National Institute of Health Stroke Scale; R, right; SD, standard deviation; WAB-AQ, Western Aphasia Battery-Aphasia Quotient; WAIS, Wechsler Adult Intelligence Scale; W, White; y, year.

### Assessment of language and other cognitive measures

All participants underwent a battery of language and neuropsychological testing at the time of enrolment. We focused our analyses on the four subtests of the WAB-R^41^: spontaneous speech (SS), repetition (R), naming (N), and auditory comprehension (AC). Spontaneous speech is rated on a 20-point scale based on information content and fluency in conversational speech and picture description. Repetition evaluates the ability to repeat auditorily presented words and sentences of increasing complexity on a 10-point scale. The naming subtest assesses picture naming, word fluency, and sentence completion on a 10-point scale. Finally, the auditory comprehension subtest includes auditorily presented yes/no questions, single word, and sentence comprehension and is assessed on a 10-point scale.

In the remaining neuropsychological battery, participants completed the Wechsler Adult Intelligence Scale-IV Matrix Reasoning subtest (WAIS)^44^ and Apraxia of Speech Rating Scale (ASRS),^45^ which were used to ensure that the participants did not exhibit significant cognitive deficits or apraxia of speech beyond the stroke-related language impairment. The test battery was administered by American Speech-Language-Hearing Association (ASHA)–certified speech-language pathologists with experience working with individuals with aphasia.

### Acquisition of neuroimaging data

All participants underwent research MRI scanning on Siemens Trio 3T scanners equipped with a 20-channel head coil, located at the University of South Carolina or at the Medical University of South Carolina. The following sequences were obtained from all participants:

- T1-weighted (MP-RAGE) sequence with 1 mm isotropic voxels, a 256 × 256 matrix size, a 9° flip angle and a 92-slice sequence with repetition time (TR) = 2250 ms, inversion time (TI) = 925 ms and echo time (TE) = 4.11 ms.
- T2-weighted scans were acquired using a 3D T2-weighted SPACE sequence covering the whole head with a resolution of 1 mm^3^ and a field of view = 256 × 256 mm, 160 sagittal slices, variable degree flip angle, TR = 3200 ms and TE = 212 ms.
- Diffusion-weighted images (DWI) were acquired using four sequence acquisition, with two acquired in the anterior–posterior (AP) direction and two in the posterior-anterior (PA) direction. The two acquisitions were otherwise identical sequences (duration: 4:02 min) with the following settings: 1.5 mm isotropic voxels and a field of view = 210 × 210 × 120, 90° flip angle, TR = 5250 ms, TE = 80 ms, 42 dir., monopolar, slices = 80, averages = 1 and diffusion values = 2 (0 s/mm^2^, 2000s/mm^2^).
- Resting-state fMRI (rsfMRI) was acquired using a 12:30 min EPI sequence with 2.4 × 2.4 × 2.0 mm voxels and a field of view = 216 × 216 × 120, 72° flip angle, TR = 1650 ms and TE = 35 ms, with 472 volumes collected, ascending interleaved and eyes closed.
- Task-based fMRI (fMRI) was acquired using sparse T2* MRI echoplanar imaging with the following sequence parameters: 60 full brain volumes, 90° flip angle, TR = 10 s, TA = 2 s, TE = 30 ms, matrix = 64 × 64, in-plane resolution = 3.25 × 3.25 mm, slice thickness = 3.2 mm (no gap) and 33 axial slices collected in planes aligned parallel to the AP commissure line.
- ASL scans were acquired with a 4:22 min PCASL sequence with 3.0 × 3.0 × 6.0 mm voxels, 25% distance factor and a field of view = 210 × 210 × 04, 90° flip angle, TR = 2500 ms and TE = 13 ms, with GRAPPA 2 acceleration and 800 ms bolus duration.

### Pre-processing of neuroimaging data

Six broad categories of neuroimaging were derived based on the sequences described above. These categories are outlined below.

1. *Lesion characteristics*: Lesion data were binarized for LSM. Chronic stroke lesions were manually drawn using each participant’s T2-weighted image in native space. All lesion tracings were performed by an expert neurologist or by a trained study staff member who was blinded to the behavioural data. Enantiomorphic normalization was conducted using the Clinical Toolbox. This process leverages SPM12’s unified segmentation-normalization method to warp this chimeric image to standard Montreal Neurological Institute (MNI)^46^ space, and the resulting spatial transform was then applied to the native-space non-healed T1 scans as well as the native-space versions of the hand-drawn lesion map. The normalized T1 created in this step was used to create probabilistic maps of lesion location using publicly available code included in the *nii_preprocess* repository (i.e. *nii_i3 m.m*).

**Figure 1:**
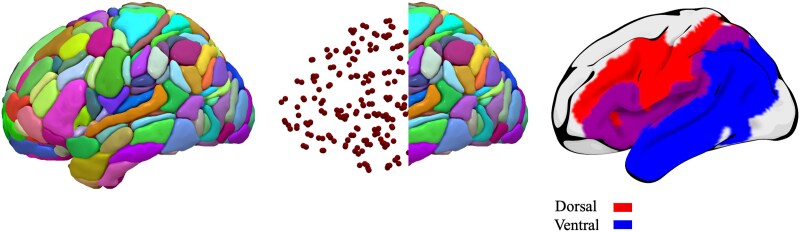
**AICHA^47^ and dual streams of speech processing.^4,5^** The dorsal speech stream is visualized in red, the ventral speech stream is visualized in blue and overlapping regions depicted in purple.

We measured the overlap between the lesion mask in standard space and each homotopic region of interest (ROI) in the Atlas of Intrinsic Connectivity of Homotopic Areas (AICHA; Fig. 1)^47^ to define the regional distribution of stroke-related damage. AICHA has 192 homotopic regions in each hemisphere (384 total areas), and the result of this approach was an n*m vector, where *n* is the number of participants (86), *m* is the number of regions (384), and each *n,m* entry corresponded to the percentage of damage of region m for individual *n*.

We also employed a separate automatic approach using probabilistically defined maps of lesion location estimated directly from T1-weighted images, entitled i3mT1. i3mT1 uses a combination of thresholding, smoothing, and masking to detect lesioned areas (relatively dark on a T1 and relatively bright on a T2) in a normalized T1-weighted MRI scan and results in a probabilistic, as opposed to binary lesion mask.^48^ As a result, a whole brain image containing voxel-wise lesion probabilities was obtained in MNI space. Similarly to the manual approach for lesion estimation described above, the i3mT1 image was co-registered with the AICHA atlas, yielding an 86*384 matrix, where each *n,m* entry corresponded to the likelihood of damage of region m for individual *n*.

2) *Connectomics data*: Connectomics refers to both functional and structural connectomes optimized for post-stroke connectome LSM (CLSM) from high-resolution diffusion tensor imaging tractography and resting-state fMRI. DWI-imaging-based structural connectivity was estimated using the *nii_preprocess* pipeline. Briefly, FSL’s EDDY was used to correct for eddy currents and participant movement, and TOPUP was used to correct for susceptibility-induced distortion. Fibre tractography for each region in the AICHA atlas was performed on the resulting images using the PROBTRACKX tool and the number of streamlines connecting each area. This resulted in a 384*384*n matrix of streamline counts representing the strength of structural connectivity between each pair of brain areas for each participant. Resting-state fMRI (rsfMRI) data were also processed using standard lab procedures outlined in the *nii_preprocess* pipeline, including using SPM12 realign and unwarp with default settings, brain extraction, slice timing correction, realignment with the T1-weighted image, normalization into MNI space based on the transformation matrix described above, band-pass filtering of the data (0.01–0.1 Hz), and, finally, application of independent component analysis (ICA) to remove lesion-driven artefacts as described in prior work.^49^ The correlation between signal strength in each pair of AICHA regions was calculated, resulting in a 384*384*n matrix of functional connectivity data. In addition to pairwise functional connections, rsfMRI data were used to extract partial amplitude of low frequency fluctuations (pALF). pALF was computed as described by Zou *et al*.^50^: Signal amplitude in each ROI (limited to frequencies between 0.01 Hz and 0.08 Hz) was divided by the summed signal amplitude of all frequencies in that same ROI. The values generated using this approach can be interpreted as representing the strength of relevant (i.e. non-noise) spontaneous brain activity within each specific brain area.
3) *VBM*: VBM was computed using the Cat12 toolbox, Ver. 12.7, with default settings, using the enantiomorphically healed T1-images (described above) as input. This processing step resulted in the generation of cortical GM volume and WM volume values for each ROI in the AICHA atlas. These values were normalized by total intracranial volume (TIV) in order to account for differences in brain size, and the resulting 384*n matrix was used in subsequently described analyses.
4) *Task-based functional MRI (fMRI)*: Participants completed a picture-naming paradigm in the scanner.^51,52^ Briefly, participants were asked to name 40 object images and stay silent during the presentation of 20 abstract images. Standard pre-processing was applied to sparse fMRI data collected during object naming. Following motion correction, realignment, normalization, and smoothing, SPM12 was used to create a GLM modelling data acquired during naming of real noun pictures and silent viewing of abstract pictures. Following model estimation, a contrast image representing the comparison ‘real > abstract’ was generated. Average signal observed for this contrast was calculated for each region of the AICHA atlas, resulting in a 384*n matrix.
5) *Fractional anisotropy (FA) and mean diffusivity (MD)*: FA and MD values were obtained from DTI data (following application of TOPUP and EDDY as described above), using FSL’s DTIFIT.^53^ FA and MD values were extracted for each region of the AICHA atlas resulting in two 384*n matrices, respectively.
6) *Cerebral blood flow (CBF)*: CBF data were generated using ASL images and the ASLtbx.^54^ The first volume of the ASL data was spatially aligned to the T1-weighted image, and this transform was applied to all processed ASL images, which were then normalized into standard MNI space. Mean cerebral blood flow values were calculated for each region of the AICHA atlas, resulting in a 384*n matrix.

### Data analysis

PLSR was employed to examine co-varying patterns of lesion damage and language impairment. PLSR is a multivariate statistical approach that is particularly advantageous when dealing with data sets where predictor and response variables are highly collinear and complex, as is the case in post-stroke aphasia. The core principle of PLSR lies in its ability to extract latent variables (LVs), or components, that maximize the covariance between the predictor and response variables. This is achieved through SVD, which reduces the dimensionality of the data while retaining the most significant relationships between neuroimaging features and language performance. Consistent with the study aims, separate neuroimaging modality-specific PLSR models were constructed to investigate shared variance and variance uniquely associated with specific WAB-R subtest scores.

Specifically, SVD was employed to decompose the covariance matrix between the pre-processed neuroimaging features (*X*) and WAB-R scores (*Y*), enabling the identification of LVs that captured the greatest amount of X-Y covariance. Feature loadings for the X and Y matrices were computed for 20 LVs, reflecting which original variables had the highest loadings in each LV. The resulting LVs were ranked based on explained variance to discern primary patterns of lesion anatomy associated with the observed language deficits, and the five highest-ranking LVs were retained for further analyses. To evaluate the robustness and predictive power of the identified LVs, we applied leave-one-out cross-validation (LOOCV). For each iteration of the LOOCV, a single subject was excluded from the data set, and the PLSR model was trained on the remaining data (*N*-1). The trained model was subsequently used to predict language scores for the left-out subject. Beta coefficients were computed by regressing the observed WAB-R subtest scores onto the LVs. These coefficients quantify the strength and direction of the relationship between each LV and the WAB-R subtest scores, indicating how strongly each latent component is associated with the observed language outcomes. Model accuracy was evaluated based on Pearson’s correlation coefficients between the predicted and actual WAB-R scores.

In order to distinguish neural features shared across subtest scores from those uniquely associated with each subtest, we extracted VIP scores to assess the contribution of each brain region in the PLSR models, reflecting their influence across all LVs, and beta coefficients characterizing the relationship between LVs and each subtest score. Specifically, VIP scores were derived by summarizing the influence of each feature across all modality-specific LVs, weighted by the variance explained by each LV in predicting WAB-R scores. Higher VIP scores indicate features that are more important for the model’s ability to predict language outcomes, and a VIP threshold of 1.5 was applied to identify the most significant features, ensuring the interpretability of the most influential brain–behaviour relationships.^55-57^ The highest-ranking VIP brain regions identified within and across modalities provide a clear ranking of the regions most strongly associated with WAB-R performance, thus representing shared neuroanatomical substrates associated with the observed language impairments. Conversely, unique features were defined as brain regions with strong beta coefficients for a specific subtest but low or negligible coefficients for other subtests, suggesting distinct neural substrates for a given language function. Beta values were reshaped to align with the structure of the neuroimaging features and a threshold of ±0.2 was applied to identify regions associated with WAB-R subtest scores. Beta values were subsequently rescaled (range 0–1) to enable direct comparison across neuroimaging modalities. Regions with beta values exceeding the threshold for only one subtest but not the other three were considered uniquely associated with that subtest. Crucially, this approach allowed for the differentiation of brain regions and networks with broad or specialized roles in post-stroke language deficits from a multimodal perspective.

Importantly, the multimodal neuroimaging approach applied here allowed us to characterize the effects of lesion damage on language function from multiple perspectives. Since each modality-specific PLSR model was constructed independently, the results demonstrate latent brain–behaviour relationships as captured by different neuroimaging modalities (e.g. lesion versus structural connectivity versus perfusion). While the complementarity of multimodal over unimodal LSM in aphasia remains a topic of debate,^24,26,58-60^ this approach highlights how different imaging modalities can uncover both common and distinct neural correlates of language deficits, providing a comprehensive view of the neural substrates involved. As such, the reported findings lay the groundwork for future research aiming to improve the prediction of language outcomes by integrating multiple neuroimaging modalities, thereby advancing the understanding and treatment of post-stroke aphasia.

All PLSR estimations were conducted using the *plsregress* function in MATLAB and LOOCV methods, and statistical analyses were performed using custom MATLAB scripts.

## Results

**Figure 2:**
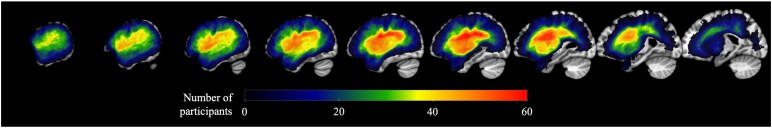
**Lesion overlap across participants.** Maximum overlap (*N* = 65 participants) was observed in the superior longitudinal fasciculus.

Participant characteristics are presented in Table 1; Fig. 2 shows lesion overlap in the sample. We observed substantial variability both in language outcomes (Table 2) and lesion characteristics. Highest average language scores were achieved on the auditory comprehension subtest (7.7/10 ± 1.8; 77.0% ± 18.0%), whereas similar average scores were achieved on spontaneous speech (11.4 ± 4.9; 57.0% ± 24.5%), naming (5.4 ± 3.0; 54.0% ± 30.0%) and repetition (5.2 ± 2.9; 52.0% ± 29.0%). Language scores were inter-correlated (*r* = 0.63–0.84, all *P* < 0.01) and correlated with overall severity of language impairment (WAB-AQ; *r* = 0.79–0.96, all *P* < 0.01; Table 2).

**Table 2:** Distribution of Western Aphasia Battery subtest scores

Variable	Spont. speech	Naming	Speech repetition	Auditory comprehension
Range	3.0–19.0	0.1–9.6	0.1–10.0	3.0–10.0
Mean	11.4	5.4	5.2	7.7
SD	4.9	3.0	2.9	1.8
Pearson’s *r*				
Spont. speech		0.84**	0.84**	0.68**
Naming			0.83**	0.74**
Speech rep.				0.63**
Aud. comp.				
WAB-AQ	0.96**	0.94**	0.92**	0.79**

Pearson’s correlation coefficient (*r*) was calculated for each pair of measures.

SD, standard deviation; WAB-AQ, Western Aphasia Battery-Aphasia Quotient. *P < 0.05; **P < 0.01.

The heterogeneity in patterns of language deficits, correlation between scores on different WAB-R subtests and variable lesion characteristics emphasize the utility of a multivariable modelling approach like PLSR to inform salient neural and language factors.

### Latent decomposition of the brain–behaviour covariance

**Figure 3:**
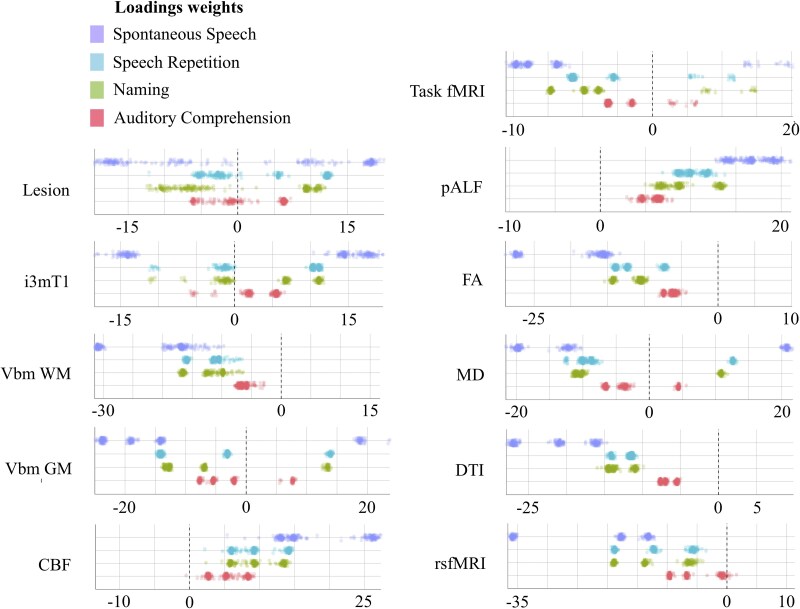
**Latent decomposition of WAB-R subtest scores.** Each dot represents the relative contribution of subscores across *N*-1 LOOCV folds (overall *N* = 86). CBF, cerebral blood flow; DTI, diffusion tensor imaging; FA, fractional anisotropy; fMRI, functional magnetic resonance imaging; GM, grey matter; MD, mean diffusivity; pALF, perfusion amplitude of low-frequency fluctuations; rsfMRI, resting-state functional magnetic resonance imaging; VBM, voxel-based morphometry; WM, white matter.

PLSR was applied to decompose the shared covariance between neuroimaging features and language performance across the four subtests of the WAB-R. Figure 3 demonstrates the relative loadings of the four WAB-R subtests in the decomposed brain–behaviour covariance matrix. The spontaneous speech subtest emerged as a dominant component in all PLSR models. The loadings of the naming and repetition subtests were comparable in the structural connectivity, functional connectivity, fMRI, pALF, VBM WM, CBF, FA and MD models; repetition had greater absolute loading weight in the Lesion and i3mT1 models, whereas naming contributed more in the VBM GM model. The auditory comprehension subtest consistently had the lowest absolute loading weight across all models.

**Figure 4:**
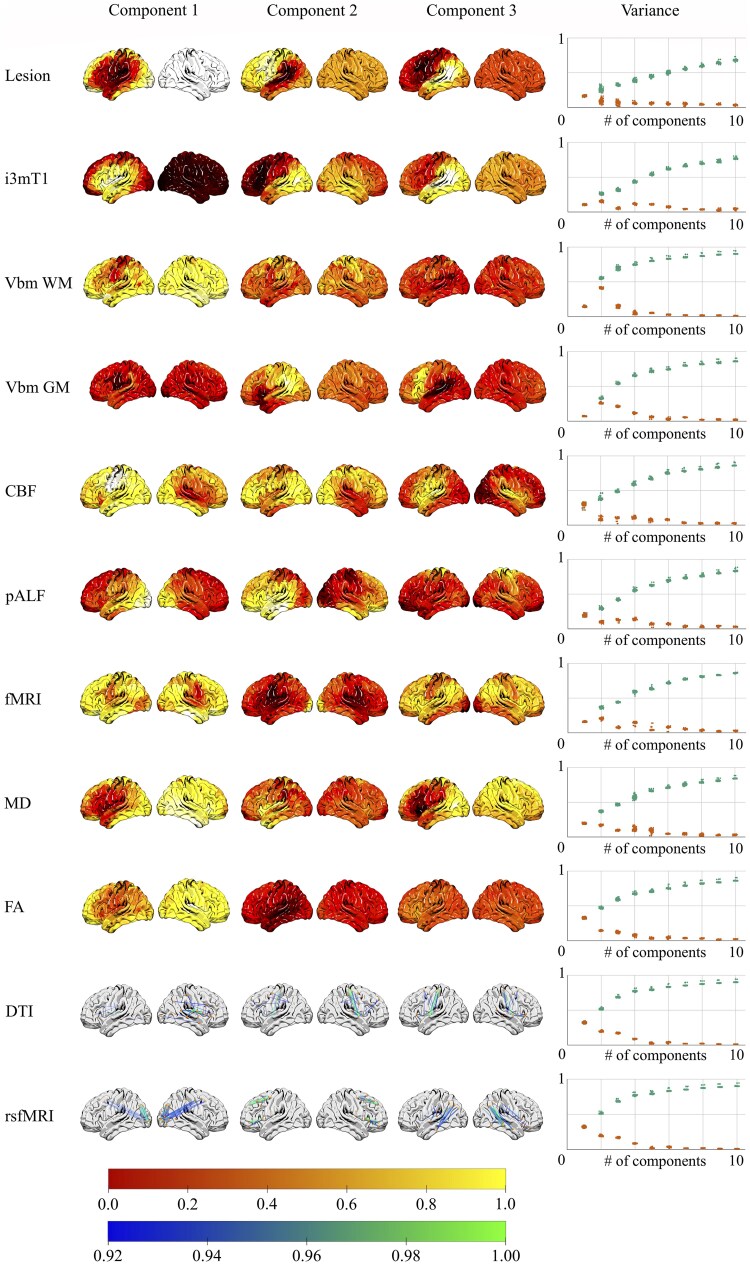
**Latent decomposition of neuroimaging measures.** Brain renderings represent the first three neuroimaging components accounting for the greatest amount of variance in the brain-language covariance matrix (overall *N* = 86). Corresponding plots show component-specific and cumulative variance explained across models. Colour bars reflect regional loading weights. CBF, cerebral blood flow; DTI, diffusion tensor imaging; FA, fractional anisotropy; fMRI, functional magnetic resonance imaging; GM, grey matter; MD, mean diffusivity; pALF, perfusion amplitude of low-frequency fluctuations; rsfMRI, resting-state functional magnetic resonance imaging; VBM, voxel-based morphometry; WM, white matter.

Figure 4 illustrates the loading weights reflected in the first three LVs derived from the neuroimaging modality-specific PLSR models. The proportion of brain–behaviour covariance explained by the LVs varied considerably across models. Models based on VBM of WM, as well as functional and structural connectivity, explained over 50% of the brain–behaviour covariance relying only on the first two latent variables. These models emphasize a network-wide patterns of disconnections predominantly affecting the left hemisphere, including pre- and post-central gyri, insula and superior temporal gyrus, alongside preserved connectivity in networks terminating in the left and right temporo-occipital regions.

For other modalities, achieving a comparable explanatory power of the brain–behaviour covariance required three (VBM GM, CBF, FA), four (pALF, fMRI, MD), five (i3mT1) or six (Lesion) LV components. Despite differences in the number of LVs needed, common patterns emerged across modalities. The first LV component consistently highlighted the left perisylvian area, encompassing strong loadings of the precentral gyrus, superior temporal gyrus and insula. Perfusion and connectivity models also identified significant involvement of right hemisphere homologous regions, reflecting bilateral contributions to language processing.

The second LV component largely delineated the ventral and dorsal streams, with high loadings in the middle and posterior temporal lobes, indicating their critical roles in both lesion and connectivity models. The third LV component further accentuated the AP distinction, particularly in regions such as the posterior temporal lobe, central sulcus and inferior temporal gyri, with bilateral involvement in some modalities. A detailed overview of regions implicated in each component across models is presented in the Supplementary material (Supplementary Tables 1–10).

Across all models, commonalities and distinctions were observed in the involvement of specific brain regions. For instance, the structural connectivity models highlighted disconnections within the left perisylvian network, while functional connectivity models revealed more distributed network alterations, particularly in the temporo-occipital regions. This nuanced view of brain–behaviour relationships highlights the complementary insights provided by different neuroimaging modalities and underscores the importance of integrating multiple perspectives to fully understand the neural underpinnings of language function.

### Predicting language deficits from shared lesion characteristics

The primary aim of the current study was to distinguish shared lesion characteristics associated with language deficits and lesion characteristics uniquely associated with performance on a given WAB-R subtest. To assess the degree to which language deficits emerge from damage to shared neural architecture, we examined the simultaneous projection of WAB-R subtest scores onto neuroimaging modality-specific LV components. The importance of specific brain regions was derived based on the regions’ VIP ranking, i.e. the relative contribution of each region in accounting for brain–behaviour covariance across all LVs. In order to minimize the risk of overfitting the data and to ensure interpretability, we restricted the regression analysis to the first five LVs and the regions with the highest-ranking VIP scores (*N*_VIP_ = 50 for structural and functional connectivity models; *N*_VIP_ = 30 for all other modalities; all VIP > 1.5).

**Figure 5:**
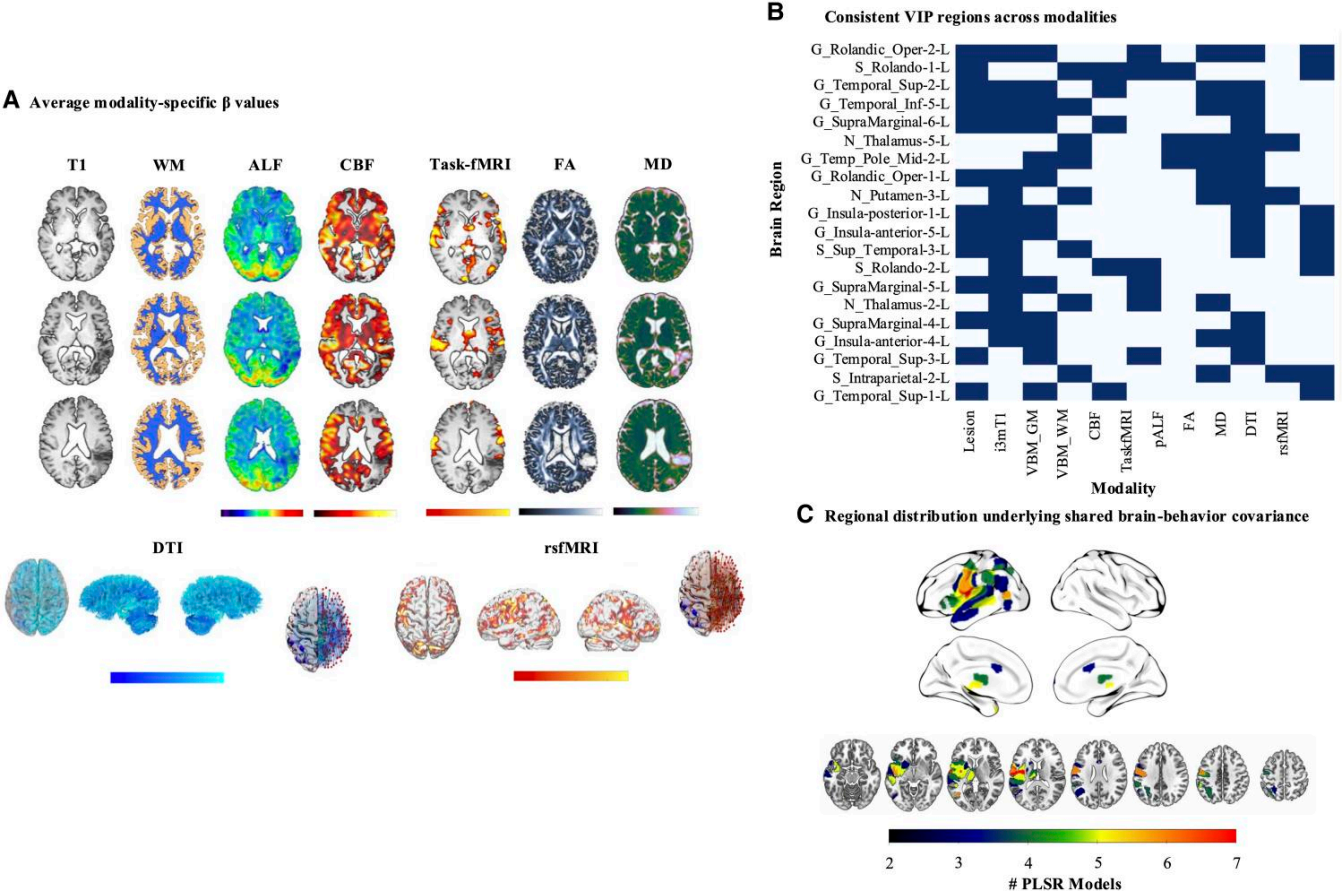
**Brain regions accounting for shared variance across WAB-R subtests.** Brain regions were ranked based on VIP scores in each modality-specific PLSR model and regions involved in at least four models are visualized (overall *N* = 86). (**A**) Mean beta coefficients associated with the projection of WAB-R subtest scores onto the latent brain–behaviour components (colour scales represent association strength); (**B**) regions demonstrating VIP scores > 1.5 in four (lower bound) to seven (upper bound) models (highlighted cells indicate involvement); (**C**) anatomical mapping of the primary VIP regions underlying shared brain–behaviour covariance. CBF, cerebral blood flow; DTI, diffusion tensor imaging; FA, fractional anisotropy; fMRI, functional magnetic resonance imaging; GM, grey matter; MD, mean diffusivity; pALF, perfusion amplitude of low-frequency fluctuations; rsfMRI, resting-state functional magnetic resonance imaging; VBM, voxel-based morphometry; VIP, variable importance in projection; WAB-R, Western Aphasia Battery-Revised; WM, white matter.

Across modality-specific models, the left rolandic operculum emerged as the most frequently implicated region, identified in seven models (Fig. 5; Supplementary Fig. 1). Other perisylvian regions in the left hemisphere were implicated in six modalities, including the superior and inferior temporal gyri. Notably, in addition to insular and temporo-parietal areas, subcortical regions such as the putamen and thalamus were identified as critical regions in four to five modalities, underscoring their involvement in language processing. The pattern of findings across different neuroimaging modalities reveals important insights into the neurobiology of language impairment in aphasia. Structural connectivity models (e.g. DTI) highlighted disconnections in key language regions such as the superior temporal gyrus, whereas lesion-based modalities (e.g. Lesion and i3mT1) implicated direct damage to areas like the rolandic operculum. In contrast, modalities assessing functional activity and perfusion (e.g. rsfMRI, task-fMRI, and CBF) highlighted the involvement of both left and right hemisphere homologous regions, reflecting network-wide disruptions beyond the lesion site.

**Figure 6:**
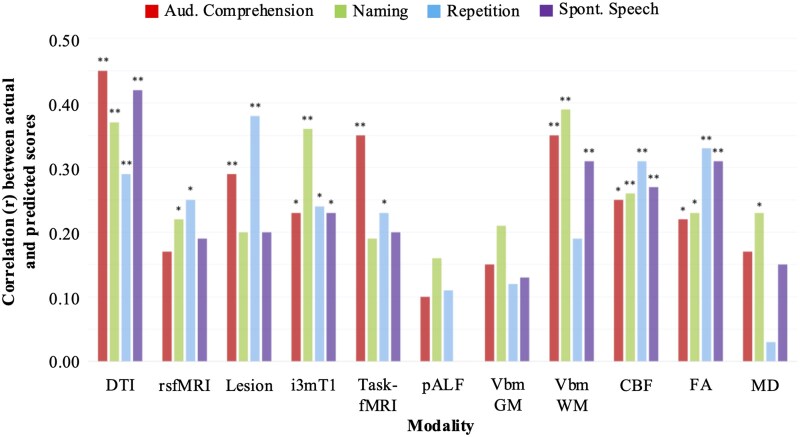
**Regression estimates relating WAB-R subtest scores to modality-specific VIP regions.** The bar plot demonstrates prediction accuracy for each WAB-R subtest based on Pearson’s correlation coefficient (*r*) between actual and predicted outcomes (overall *N* = 86). CBF, cerebral blood flow; DTI, diffusion tensor imaging; FA, fractional anisotropy; fMRI, functional magnetic resonance imaging; GM, grey matter; MD, mean diffusivity; pALF, perfusion amplitude of low-frequency fluctuations; rsfMRI, resting-state functional magnetic resonance imaging; VBM, voxel-based morphometry; WM, white matter. *P < 0.05; **P < 0.01.

**Figure 7:**
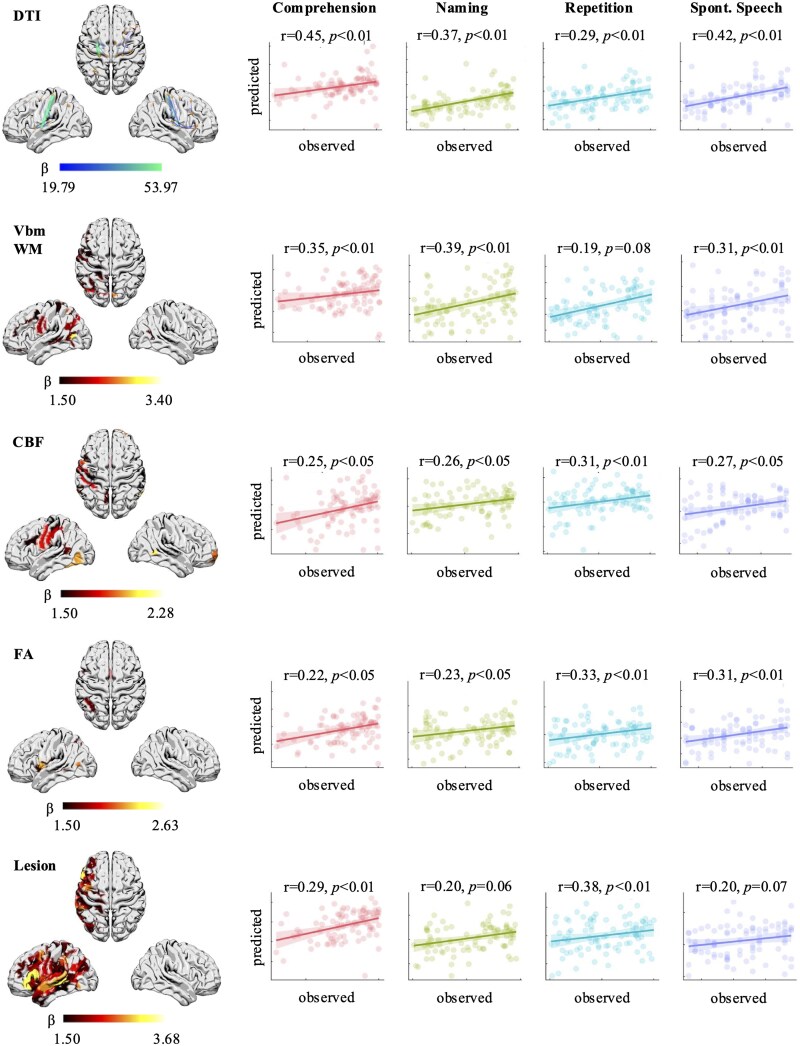
**VIP regions and regression estimates.** VIP and prediction results for the five models yielding the most accurate prediction across WAB-R subtest scores (overall *N* = 86). The scatter plots demonstrate the correlation between actual (*x*-axis) and predicted (*y*-axis) WAB-R subtest scores. CBF, cerebral blood flow; DTI, diffusion tensor imaging; FA, fractional anisotropy; fMRI, functional magnetic resonance imaging; GM, grey matter; MD, mean diffusivity; pALF, perfusion amplitude of low-frequency fluctuations; rsfMRI, resting-state functional magnetic resonance imaging; VBM, voxel-based morphometry; VIP, variable importance in projection; WM, white matter.

WAB-R subtest scores were subsequently regressed on the highest-ranking regions within each modality to determine the extent to which shared lesion anatomy accounts for variance in language performance. Model performance was evaluated based on Pearson’s correlation between actual and LOOCV-predicted language outcomes. A statistically significant correlation between actual and predicted scores was observed in 26/44 models (Fig. 6). Several modalities yielded significant results for all four language functions, including structural connectivity (DTI), i3mT1, CBF, and FA, whereas the pALF and VBM GM models did not yield any significant results (Fig. 7; Supplementary Figs 2–4). The structural connectivity model yielded the highest correlation coefficients for auditory comprehension (*r* = 0.45, *P* < 0.01) and spontaneous speech (*r* = 0.42, *P* < 0.01); naming was most accurately predicted by the VBM WM model (*r* = 0.39, *P* < 0.01); and repetition was most accurately predicted based on structural Lesion data (r = 0.38, *P* < 0.01).

### Neural architecture uniquely associated with specific language deficits

**Figure 8:**
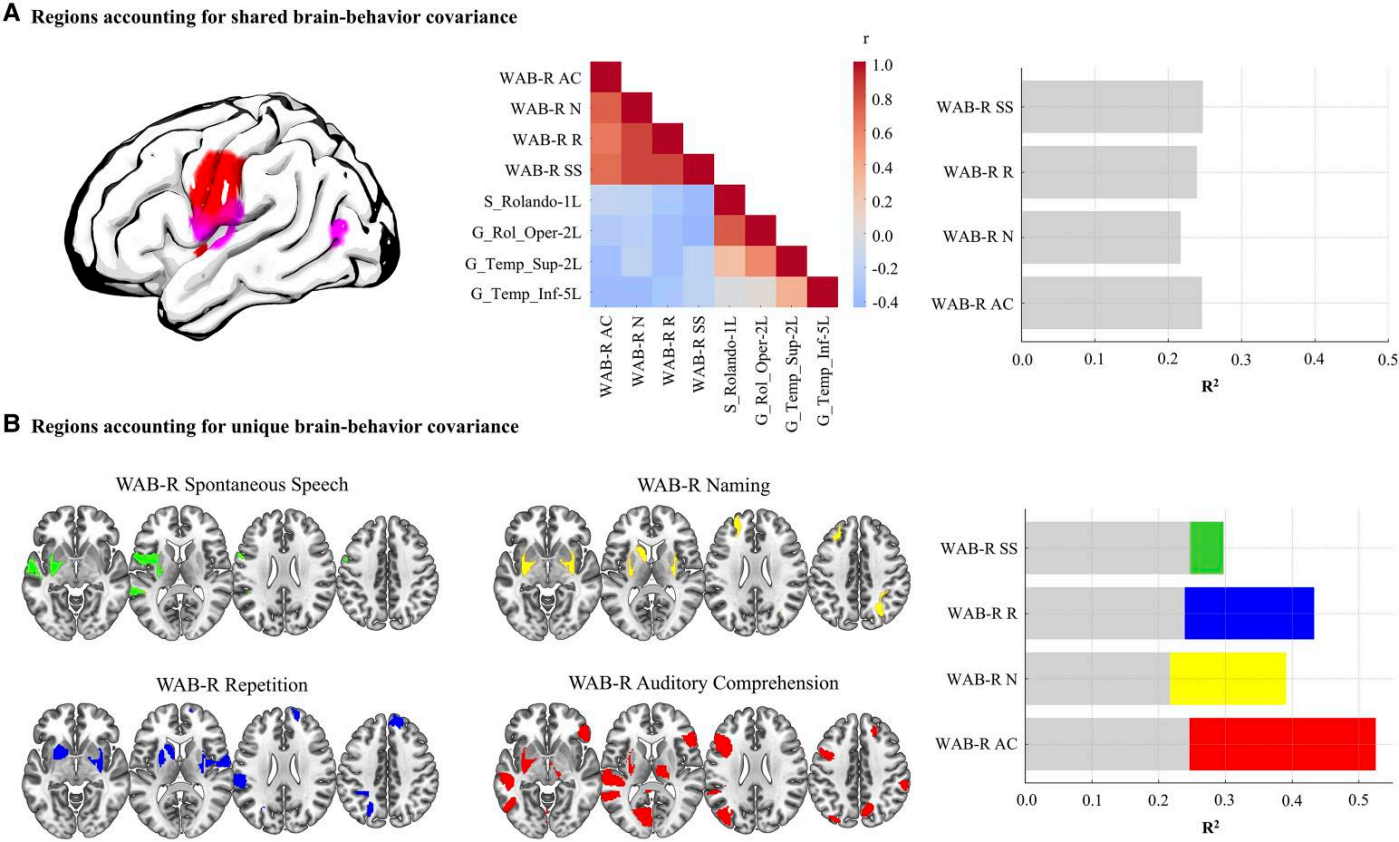
**Brain regions accounting for shared and unique variance in language.** (**A**) Brain regions accounting for the greatest amount of shared variance in WAB-R subtest scores (colour reflects regional boundaries). The heatmap demonstrates Pearson’s correlation coefficient (*r*) between proportional lesion to brain regions associated with shared variance in at least six neuroimaging modality-specific PLSR models and WAB-R subtest scores (overall *N* = 86). The bar plot visualizes amount of variance explained (*R*^2^) by regressing WAB-R subtest scores on proportional lesion to the same four brain regions; (**B**) brain regions accounting for unique residual variance in WAB-R subtest scores. Unique regional involvement was determined based on a threshold of averaged ß=±0.2 for a given WAB-R subtest and lower coefficient for other subtests (overall *N* = 86). The bar plot visualizes amount of variance explained (*R*^2^) by regressing WAB-R subtest scores on proportional lesion to the same four ‘shared’ regions in addition to mean diffusivity values in regions associated with ‘unique’ variance across WAB-R subtests. AC, auditory comprehension; N, naming; R, repetition; SS, spontaneous speech; WAB, Western Aphasia Battery-Revised.

The shared lesion analysis revealed that damage to areas immediately surrounding the perisylvian fissure and the posterior-temporal junction accounted for the greatest amount of shared variability in post-stroke language function. The heatmap presented in Fig. 8 demonstrates the relationship between damage in these regions and impaired language function across all four WAB-R subtests (*r* = −0.15 to −0.43). Consistent with the second study aim, we subsequently identified brain regions uniquely associated with specific WAB-R subtests. Following the PLSR model fitting for each neuroimaging modality, regions with high beta values for one subtest but low or negligible coefficient for others were classified as uniquely contributing to that specific subtest. Positive and negative beta coefficients were scaled to enable direct comparison across neuroimaging modalities (e.g. lesion in a given region can impair performance, whereas perfusion in the same region may facilitate performance), such that a significant effect indicates regional involvement for a given subtest rather than the direction of the effect. In order to prevent spurious findings, we report regional task associations identified in two or more modality-specific PLSR models.

A widespread network of regions was uniquely associated with performance on the auditory comprehension subtest, primarily highlighting posterior occipital (e.g. calcarine gyri), inferior parietal and middle and inferior temporal lobe regions, as well as subcortical regions involved in cortical projections (e.g. putamen, thalamus). Although most regions were left lateralized, several regions in the right hemisphere were similarly implicated, including frontal, parietal and subcortical areas. Importantly, the right hemisphere associations were primarily identified in models based on connectivity, tissue microstructure and volumetric measures, whereas left hemisphere regions were more frequently identified in lesion-based models (e.g. Lesion, i3mT1). Several regions in the bilateral superior frontal and parietal lobes, as well as subcortical regions (e.g. caudate nucleus, putamen) were uniquely associated with naming performance. These associations were predominantly revealed in connectivity-based models. Similarly, performance on the repetition subtest was found to be associated with right frontal lobe regions, left inferior parietal regions and bilateral hippocampal integrity. Last, only left lateralized frontal, temporal and subcortical regions were uniquely associated with performance on the spontaneous speech subtest. A comprehensive list of regions uniquely involved in each language task is presented in the Supplementary material (Supplementary Table 11).

Finally, to demonstrate how language impairment reflects patterns of damage to shared and unique neural architecture, we regressed each WAB-R subscore on four regions accounting for the greatest amount of shared variance (identified in at least six models; i.e. rolandic operculum; G_Rolandic_Oper-2-L, S_Rolando-1-L, superior and inferior temporal gyrus; G_Temporal_Sup-2-L, G_Temporal_Inf-5-L; see Fig. 5) and associated the resulting residuals with integrity of regions identified as uniquely related to a given subtest. This *post hoc* analysis is by no means exhaustive, but rather aims to illustrate in a principled manner that language deficits in aphasia can be viewed as a combination of global lesion effects (i.e. damage to shared neural substrates affects multiple language domains) and integrity/lesion to regions uniquely subserving specific language domains. To this end, we predicted language outcomes based on proportional lesion in the four shared regions—assuming that damage leads to poorer performance—and MD in regions uniquely associated with specific subtest scores, reflecting structural integrity of each region.

As shown in Fig. 8, the shared model accounted for 22–25% of variance across WAB-R subtests, whereas the unique models accounted for a significant portion of additional variance: 5.0% in spontaneous speech, 19.4% in repetition, 17.4% in naming, and 27.9% in auditory comprehension. After accounting for shared variance, the right putamen emerged as the strongest unique predictor of naming and repetition performance (*P* = 0.013 and 0.021, respectively), and part of the left superior temporal lobe (*P* = 0.049) and the right precuneus (*P* = 0.045) emerged as the strongest predictors of auditory comprehension. No independent effects were observed for spontaneous speech performance. Together, these results dissociate the global and regional effects of lesion damage in chronic post-stroke aphasia, lending support to the notion of aphasia as a multifaceted network disorder and motivating translational initiatives to advance neurobiologically informed treatment resources in the future.

## Discussion

The current study sought to advance our understanding of the neural underpinnings of language deficits in chronic post-stroke aphasia by leveraging PLSR to explore how damage to shared and unique brain networks contributes to multiple language domains. The integration of multimodal neuroimaging—including structural MRI, diffusion-weighted imaging, functional MRI and ASL—enabled us to comprehensively map the relationship between lesion anatomy and language impairments as reflected in WAB-R subtest scores. Our findings emphasize the importance of both global network damage and damage to regions subserving specific functions in explaining aphasic language impairment, providing insights that extend beyond traditional LSM approaches.

### Application of partial least squares regression in the context of aphasia

The primary novelty of this work lies in the network assessment of aphasia using PLSR. The results from PLSR offer a simple interpretation compared to other multivariate methods, as they directly relate to the covariance between brain regions and behavioural outcomes. Moreover, in contrast to more commonly applied approaches to reduce the dimensionality of neuroimaging or behavioural data, PLSR extracts latent components that maximize covariance between the two sets of data. As such, PLSR is especially effective when dealing with multicollinearity in brain–behaviour prediction models. Furthermore, since latent brain–behaviour components are extracted sequentially, the residual variance associated with a specific dependent variable can be analysed after accounting for the global effect of lesion. Therefore, by implementing a data-driven PLSR approach and multimodal neuroimaging, our results demonstrate patterns of lesion anatomy accounting for maximal variance in language impairment, characterized as participants’ collective performance on all four WAB-R subtests, and spatial distribution of lesions accounting for residual variance specific to each functional domain.

### Global and regional lesion anatomy associated with language impairment

Our results demonstrate that damage to the left perisylvian cortex, particularly regions surrounding the rolandic operculum and superior temporal gyrus, accounts for a substantial portion of shared variance across WAB-R subtests. The perisylvian cortex has long been recognized as a key network supporting core aspects of speech production and comprehension.^5,61-66^ By extension, its central role in the global disruption of language functions was somewhat expected, corroborating prior findings suggesting that language impairments in aphasia often result from damage to regions that support multiple interrelated processes.^22-68^ Importantly, performance on the WAB-R spontaneous speech subtest emerged as a dominant behavioural component in relation to brain lesions across neuroimaging modalities (Fig. 3), likely reflecting its reliance on a wide array of language processes, including lexical retrieval, syntactic processing and speech fluency. This finding echoes prior evidence suggesting that the WAB-R is highly influenced by deficits affecting speech production and highlights the clinical relevance of spontaneous speech performance as a marker of global language impairment as well as neural network disruption.^69^

However, a key contribution of this study lies in the identification of distinct brain regions that uniquely support specific language functions. For instance, the basal ganglia and left temporo-parietal junction were uniquely associated with performance on the naming and repetition subtests. These effects fall in line with previous research indicating that subcortical structures may be crucially involved in motor planning and articulatory aspects of speech production.^70-74^ Moreover, an extensive bilateral network primarily involving posterior occipital, temporo-parietal, inferior temporal and subcortical regions was uniquely associated with performance on the auditory comprehension subtest. The distributed nature of these regional effects indicates greater bilateral neuroanatomical involvement relative to other language functions,^5^ which could explain relatively poorer accuracy in predicting performance on tasks reflecting integrity of auditory comprehension in prior publications.^21,24,58^ Thus, a comprehensive view of our results demonstrates that dissociating global network disruptions from more targeted lesion effects is essential for understanding the full complexity of post-stroke aphasia and, subsequently, refining concurrent prognostic models.

### Multimodal neuroimaging for multiple perspectives

We applied a multimodal neuroimaging approach in an effort to comprehensively examine both the influence of frank lesion and post-stroke functional neural reorganization on language function. This approach enables direct comparison of regional effects as captured by different neuroimaging modalities. For example, structural and diffusion-weighted imaging revealed the importance of GM and WM integrity for language performance, whereas resting-state and task-based fMRI captured functional connectivity disruptions often undetected in structural analyses alone. Notably, functional connectivity (rsfMRI) analyses highlighted the involvement of right hemisphere homologue regions in auditory comprehension (Fig. 8), lending support to the notion of right hemispheric compensatory neural recruitment to support language recovery.^75,76^ This finding underscores the importance of examining language reorganization in chronic aphasia from a network perspective and suggests that bilateral connectivity might be a target for future therapeutic interventions.^77,78^ Furthermore, perfusion imaging (e.g. CBF) demonstrated that hypoperfusion in left hemisphere regions—particularly in the inferior frontal and superior temporal gyri—accounted for significant portion of shared variance in language impairment, consistent with prior research showing that hypoperfusion can persist even in structurally intact regions and contribute to chronic language deficits.^79^ Therefore, since each modality-specific PLSR model was constructed based on the same set of ROIs, cross-modality comparisons serve to inform efforts to enhance prediction of language outcomes in future research.

### Clinical implications

Aphasia is a heterogeneous disorder and individual variability in language deficit profiles reigns supreme. The results of this study offer a framework for understanding how global network damage and localized lesions contribute to the complex presentation of language impairment in aphasia, which can shed light on some of the hitherto inter-individual variability in aphasic symptomology. From a clinical perspective, this framework suggests that greater disruption of shared neural resources translates to simultaneous functional deficits in multiple language domains; therefore, therapies that focus on improving both expressive and receptive language abilities could be particularly beneficial. For instance, targeting structured practice in conversational skills and auditory comprehension might strengthen residual language networks and improve overall recovery. In addition to traditional speech-language therapy, the use of non-invasive brain stimulation (NIBS) techniques, such as transcranial magnetic stimulation or transcranial direct current stimulation, could be explored to facilitate neuroplasticity and support recovery in individuals with lesions affecting these critical areas. NIBS has been shown to enhance neural activity in underperforming regions and modulate compensatory neural recruitment, including in the intact right hemisphere, to enhance language recovery.^80-82^

Furthermore, identification of distinct neural correlates uniquely subserving specific language functions offers targeted pathways for therapeutic approaches. For example, individuals with lesions affecting basal ganglia structures, some of which were uniquely associated with naming and repetition performance, may benefit from motor-based therapies like Melodic Intonation Therapy^83,84^ or constraint-induced language therapy,^85,86^ both of which emphasize intensive use of affected language functions. The individualized application of these therapy approaches, in combination with brain stimulation techniques, can potentially enhance the effectiveness of treatment by addressing both global network disruptions and specific lesion effects.

### Limitations

Some limitations should be taken into context when interpreting the findings. First, the primary purpose of the current work was to inform the core structure of lesion-behavioural relationships in post-stroke language impairments, rather than to optimize prediction accuracy. Indeed, the goal is almost the opposite, which is to define the most salient drivers of lesion-behavioural mapping instead of fine-tuning the methods to explain residual unexplained variance. Also, we adopted multiple neuroimaging modalities to capture different aspects of the lesion damage. However, we did not combine modalities within any given model to enhance the prediction of language scores. Second, language impairment was quantified based on the WAB-R only, which is a fairly coarse measure of language processing. Similar analyses carried out using other language measures could reflect a different anatomical structure related to those tests. Finally, although our findings highlight the importance of several key areas for language performance, they do not represent an exhaustive overview of regions that contribute to language functioning.

## Conclusion

The current study examined the latent underlying structure of lesion-symptom covariance in post-stroke aphasia. By combining PLSR and multimodal neuroimaging, we characterized the complex and distributed nature of language processing, highlighting key areas for future research and clinical application. Our primary findings demonstrate that language impairments arise from both global network damage and region-specific lesions, associated with behavioural variance shared across language domains and uniquely associated with specific language functions, respectively.

## Supplementary Material

fcaf246_Supplementary_Data

## Data Availability

The data that support the findings of this study and proprietary MATLAB scripts used for data analysis are available from the corresponding author upon reasonable request.
