# Supplementary material for: Partial least squares multimodal analysis of brain network correlates of language deficits in aphasia
Source: Brain Commun. 2025 Jun 19;7(4):fcaf246. doi: 10.1093/braincomms/fcaf246 (PMC12264888; doi:10.1093/braincomms/fcaf246)
Supplement: fcaf246_Supplementary_Data [file fcaf246_supplementary_data.pdf]

Supplementary Tables

First Five Neural Components for All Modalities

FA				
**Component 1**	**Component 2**	**Component 3**	**Component 4**	**Component 5**
S_Postcentral-3-R	G_Frontal_Sup-3-L	S_Orbital-2-L	S_Precentral-3-R	G_Frontal_Mid-1-L
G_Occipital_Pole-1-R	S_Precentral-2-L	G_Insula-anterior-2-L	G_Supramarginal-1-L	G_Frontal_Mid-3-L
G_Insula-posterior-1-R	S_Precentral-3-L	G_Insula-anterior-3-L	G_SupraMarginal-2-L	G_Frontal_Mid-5-L
G_Rolandic_Oper-1-R	G_Occipital_Pole-1-R	G_Temporal_Pole_Mid-2-L	G_SupraMarginal-5-L	S_Inf_Frontal-1-L
G_Rolandic_Oper-2-R	G_Occipital_Lat-1-R	S_Parietooccipital-5-L	G_SupraMarginal-7-L	S_Inf_Frontal-2-L
S_Sup_Temporal-3-R	G_Occipital_Lat-2-R	N_Caudate-1-L	G_Angular-1-L	G_Frontal_Inf_Tri-1-L
G_Temporal_Pole_Sup-2-R	G_Occipital_Lat-3-R	N_Caudate-1-R	G_Angular-2-L	S_Precentral-1-L
G_Temporal_Pole_Mid-2-R	G_Occipital_Sup-2-R	N_Caudate-2-L	G_Angular-3-L	S_Precentral-5-L
S_Anterior_Rostral-1-R	G_Occipital_Inf-1-R	N_Caudate-2-R	G_Parietal_Inf-1-L	G_Insula-anterior-2-L
G_Frontal_Med_Orb-2-L	G_Temporal_Inf-4-R	N_Caudate-3-L	S_Intraoccipital-1-L	G_Insula-anterior-3-L
G_Frontal_Med_Orb-2-R	G_Supp_Motor_Area-2-L	N_Caudate-4-L	G_Occipital_Sup-1-L	G_Insula-anterior-4-L
G_Cingulum_Mid-1-L	G_Supp_Motor_Area-3-L	N_Caudate-4-R	G_Occipital_Mid-1-L	G_Rolandic_Oper-1-L
G_Cingulum_Post-2-L	S_Cingulate-5-L	N_Caudate-5-L	G_Occipital_Mid-2-L	G_Rolandic_Oper-2-L
G_Precuneus-4-L	G_Cingulum_Mid-2-L	N_Caudate-6-L	G_Occipital_Mid-3-L	S_Cingulate-2-L
G_Precuneus-8-R	G_Cingulum_Mid-2-R	N_Caudate-6-R	G_Temporal_Sup-3-L	S_Cingulate-5-L
S_Parietooccipital-1-L	G_Cingulum_Mid-3-L	N_Caudate-7-L	G_Temporal_Sup-4-L	G_Cingulum_Mid-1-L
G_Calcarine-1-L	G_Cingulum_Post-1-L	N_Caudate-7-R	S_Sup_Temporal-3-L	G_Cingulum_Mid-1-R
G_Calcarine-1-R	G_Cingulum_Post-1-R	N_Putamen-2-L	S_Sup_Temporal-4-L	G_Cingulum_Mid-2-L
G_Calcarine-2-R	G_Cingulum_Post-2-L	N_Thalamus-1-L	S_Sup_Temporal-5-L	G_Cingulum_Mid-2-R
G_Lingual-2-L	G_Cingulum_Post-3-L	N_Thalamus-2-L	G_Temporal_Mid-2-L	G_Cingulum_Mid-3-L
G_Lingual-5-R	G_Cingulum_Post-3-R	N_Thalamus-4-L	G_Temporal_Mid-3-L	N_Amygdala-1-L
G_Hippocampus-1-R	G_Precuneus-1-R	N_Thalamus-5-R	G_Temporal_Mid-4-L	N_Caudate-6-L
G_ParaHippocampal-2-R	G_Cuneus-1-R	N_Thalamus-6-L	G_Temporal_Inf-5-L	N_Pallidum-1-L
G_Fusiform-4-R	G_Cuneus-2-R	N_Thalamus-6-R	G_Cingulum_Mid-1-L	N_Pallidum-1-R
G_Fusiform-6-R	G_Calcarine-3-R	N_Thalamus-7-L	G_Cingulum_Post-2-L	N_Putamen-2-L
G_Fusiform-7-R	G_Lingual-2-R	N_Thalamus-7-R	G_Cingulum_Post-2-R	N_Putamen-3-L
N_Pallidum-1-R	G_Lingual-5-R	N_Thalamus-8-L	G_Cingulum_Post-3-L	N_Thalamus-2-L
N_Putamen-2-R	G_Fusiform-7-R	N_Thalamus-8-R	G_Cingulum_Post-3-R	N_Thalamus-3-L
N_Putamen-3-R	N_Pallidum-1-R	N_Thalamus-9-R	G_Precuneus-1-R	N_Thalamus-5-L

Supplementary Table 1. Component Loadings of the Fractional Anisotropy (FA) Partial Least Squares Model for Language Outcomes.

i3mT1				
**Component 1**	**Component 2**	**Component 3**	**Component 4**	**Component 5**
S_Inf_Frontal-2-L	G_Angular-3-L	G_Supramarginal-1-L	G_subcallosal-1-L	S_Precentral-2-L
G_Frontal_Inf_Tri-1-L	G_Occipital_Lat-1-L	G_SupraMarginal-2-L	G_Cingulum_Mid-2-L	S_Precentral-4-L
S_Precentral-1-L	G_Occipital_Lat-2-L	G_Supramarginal-4-L	G_Cingulum_Mid-2-R	S_Precentral-5-L
S_Rolando-1-L	G_Occipital_Lat-4-L	G_SupraMarginal-5-L	G_Cingulum_Post-1-L	S_Rolando-1-L
S_Postcentral-1-L	G_Occipital_Lat-5-L	G_SupraMarginal-6-L	G_Hippocampus-1-L	S_Rolando-2-L
G_Supramarginal-1-L	G_Occipital_Mid-1-L	G_SupraMarginal-7-L	G_Hippocampus-2-L	S_Rolando-3-L
G_SupraMarginal-2-L	G_Occipital_Mid-2-L	G_Angular-1-L	N_Caudate-1-L	S_Rolando-4-L
G_Insula-anterior-2-L	G_Occipital_Mid-3-L	G_Angular-2-L	N_Caudate-1-R	S_Postcentral-1-L
G_Insula-anterior-3-L	G_Temporal_Sup-3-L	G_Angular-3-L	N_Caudate-2-L	S_Postcentral-2-L
G_Insula-anterior-4-L	S_Sup_Temporal-3-L	G_Occipital_Mid-1-L	N_Caudate-2-R	S_Postcentral-3-L
G_Insula-anterior-5-L	S_Sup_Temporal-4-L	G_Occipital_Mid-2-L	N_Caudate-3-L	G_Parietal_Sup-1-L
G_Insula-posterior-1-L	S_Sup_Temporal-5-L	G_Occipital_Mid-3-L	N_Caudate-4-L	G_Parietal_Sup-3-L
G_Rolandic_Oper-1-L	G_Temporal_Mid-3-L	G_Occipital_Mid-4-L	N_Caudate-4-R	G_Parietal_Sup-4-L
G_Rolandic_Oper-2-L	G_Temporal_Mid-4-L	G_Occipital_Inf-1-L	N_Caudate-5-L	G_Supramarginal-1-L
G_Temporal_Sup-1-L	G_Temporal_Inf-5-L	G_Temporal_Sup-2-L	N_Caudate-5-R	G_SupraMarginal-2-L
G_Temporal_Sup-2-L	S_Parietooccipital-4-L	G_Temporal_Sup-3-L	N_Caudate-6-L	G_Supramarginal-3-L
G_Temporal_Sup-3-L	G_Calcarine-2-L	G_Temporal_Sup-4-L	N_Caudate-6-R	G_Supramarginal-4-L
S_Sup_Temporal-2-L	G_Lingual-5-L	S_Sup_Temporal-2-L	N_Caudate-7-L	G_SupraMarginal-5-L
S_Sup_Temporal-3-L	G_Hippocampus-2-L	S_Sup_Temporal-3-L	N_Caudate-7-R	G_SupraMarginal-6-L
G_Temporal_Mid-1-L	G_Fusiform-6-L	S_Sup_Temporal-4-L	N_Thalamus-1-L	G_Angular-1-L
G_Temporal_Pole_Sup-1-L	G_Fusiform-7-L	S_Sup_Temporal-5-L	N_Thalamus-1-R	G_Parietal_Inf-1-L
G_Temporal_Pole_Sup-2-L	N_Caudate-1-R	G_Temporal_Mid-1-L	N_Thalamus-2-L	S_Intraparietal-1-L
G_Temporal_Pole_Mid-2-L	N_Caudate-2-R	G_Temporal_Mid-2-L	N_Thalamus-2-R	S_Intraparietal-2-L
N_Caudate-4-L	N_Caudate-4-R	G_Temporal_Mid-3-L	N_Thalamus-3-L	S_Intraparietal-3-L
N_Caudate-5-L	N_Caudate-6-R	G_Temporal_Mid-4-L	N_Thalamus-3-R	G_Occipital_Pole-1-L
N_Caudate-6-L	N_Caudate-7-R	G_Temporal_Inf-2-L	N_Thalamus-4-L	G_Insula-posterior-1-L
N_Caudate-7-L	N_Thalamus-2-R	G_Temporal_Inf-3-L	N_Thalamus-4-R	S_Cingulate-6-L
N_Putamen-2-L	N_Thalamus-6-L	G_Temporal_Inf-4-L	N_Thalamus-6-L	S_Cingulate-7-L
N_Putamen-3-L	N_Thalamus-6-R	G_Temporal_Inf-5-L	N_Thalamus-7-L	G_Paracentral_Lobule-1-L

Supplementary Table 2. Component Loadings of the i3mT1 Partial Least Squares Model for Language Outcomes.

Lesion				
**Component 1**	**Component 2**	**Component 3**	**Component 4**	**Component 5**
G_Insula-anterior-1-R	S_Sup_Frontal-4-L	G_Supramarginal-1-L	S_Sup_Frontal-4-L	G_Frontal_Inf_Tri-1-L
S_Anterior_Rostral-1-R	S_Sup_Frontal-5-L	G_SupraMarginal-2-L	S_Sup_Frontal-5-L	G_Occipital_Inf-1-L
G_subcallosal-1-R	S_Sup_Frontal-6-L	G_Supramarginal-4-L	G_Frontal_Mid-1-L	G_Insula-anterior-2-L
G_Cingulum_Ant-1-R	G_Frontal_Mid-3-L	G_SupraMarginal-5-L	G_Frontal_Mid-3-L	G_Insula-anterior-3-L
G_Lingual-6-L	G_Frontal_Mid-4-L	G_SupraMarginal-6-L	G_Frontal_Mid-4-L	G_Insula-anterior-4-L
N_Caudate-3-R	G_Frontal_Mid-5-L	G_SupraMarginal-7-L	G_Frontal_Mid-5-L	G_Insula-anterior-5-L
N_Thalamus-3-R	S_Inf_Frontal-2-L	G_Angular-1-L	S_Precentral-4-L	G_Rolandic_Oper-1-L
N_Thalamus-6-L	G_Frontal_Inf_Tri-1-L	G_Angular-2-L	S_Postcentral-2-L	G_Rolandic_Oper-2-L
N_Thalamus-9-R	S_Precentral-1-L	G_Angular-3-L	S_Postcentral-3-L	G_Temporal_Sup-1-L
	S_Precentral-2-L	G_Occipital_Mid-1-L	G_Parietal_Sup-4-L	G_Temporal_Sup-2-L
	S_Precentral-3-L	G_Occipital_Mid-2-L	G_Supramarginal-4-L	G_Temporal_Sup-4-L
	S_Precentral-4-L	G_Occipital_Mid-3-L	G_SupraMarginal-6-L	S_Sup_Temporal-1-L
	S_Precentral-5-L	G_Occipital_Mid-4-L	G_Angular-1-L	S_Sup_Temporal-2-L
	S_Precentral-6-L	G_Occipital_Inf-1-L	G_Angular-2-L	G_Temporal_Mid-1-L
	S_Rolando-1-L	G_Temporal_Sup-2-L	G_Angular-3-L	G_Temporal_Mid-2-L
	S_Rolando-2-L	G_Temporal_Sup-3-L	G_Parietal_Inf-1-L	G_Temporal_Mid-3-L
	S_Rolando-3-L	G_Temporal_Sup-4-L	S_Intraparietal-1-L	G_Temporal_Inf-1-L
	S_Rolando-4-L	S_Sup_Temporal-2-L	S_Intraparietal-2-L	G_Temporal_Inf-2-L
	S_Postcentral-2-L	S_Sup_Temporal-3-L	S_Intraparietal-3-L	G_Temporal_Inf-3-L
	G_Parietal_Sup-1-L	S_Sup_Temporal-4-L	S_Intraoccipital-1-L	G_Temporal_Inf-4-L
	G_Parietal_Sup-2-L	S_Sup_Temporal-5-L	G_Occipital_Lat-4-L	G_Temporal_Pole_Sup-1-L
	G_Insula-anterior-3-L	G_Temporal_Mid-1-L	G_Occipital_Inf-1-L	G_Temporal_Pole_Sup-2-L
	G_Insula-anterior-4-L	G_Temporal_Mid-2-L	G_Occipital_Inf-2-L	G_Temporal_Pole_Mid-1-L
	G_Rolandic_Oper-1-L	G_Temporal_Mid-3-L	G_Temporal_Mid-2-L	G_Temporal_Pole_Mid-2-L
	G_Rolandic_Oper-2-L	G_Temporal_Mid-4-L	G_Temporal_Inf-2-L	G_Hippocampus-1-L
	G_Supp_Motor_Area-2-L	G_Temporal_Inf-2-L	G_Temporal_Inf-3-L	G_Fusiform-4-L
	G_Paracentral_Lobule-2-L	G_Temporal_Inf-3-L	G_Temporal_Inf-4-L	N_Amygdala-1-L
	N_Putamen-2-L	G_Temporal_Inf-4-L	S_Parietooccipital-5-L	N_Putamen-2-L
	N_Putamen-3-L	G_Temporal_Inf-5-L	G_Fusiform-7-L	N_Putamen-3-L

Supplementary Table 3. Component Loadings of the Lesion Partial Least Squares Model for Language Outcomes.

MD				
**Component 1**	**Component 2**	**Component 3**	**Component 4**	**Component 5**
S_Orbital-1-R	S_Orbital-2-L	G_Frontal_Sup-3-R	G_Frontal_Sup-3-L	S_Postcentral-1-L
S_Orbital-2-R	S_Postcentral-3-L	S_Sup_Frontal-6-R	S_Sup_Frontal-1-L	G_Supramarginal-1-L
S_Precentral-3-L	G_Parietal_Sup-1-L	S_Precentral-3-L	S_Sup_Frontal-5-L	G_SupraMarginal-2-L
S_Intraoccipital-1-R	G_Insula-anterior-1-L	S_Precentral-3-R	S_Sup_Frontal-6-L	G_Supramarginal-3-L
G_Occipital_Lat-5-R	G_Insula-anterior-2-L	S_Precentral-6-R	G_Frontal_Mid-5-L	G_Supramarginal-4-L
G_Occipital_Inf-1-R	G_Insula-anterior-3-L	S_Rolando-4-R	S_Inf_Frontal-2-L	G_SupraMarginal-5-L
G_Temporal_Inf-1-R	G_Insula-anterior-4-L	S_Postcentral-3-R	G_Frontal_Sup_Orb-1-L	G_SupraMarginal-6-L
G_Temporal_Inf-2-R	G_Insula-anterior-5-L	G_Parietal_Sup-2-R	S_Precentral-1-L	G_SupraMarginal-7-L
G_Temporal_Pole_Mid-2-R	G_Insula-posterior-1-L	G_Angular-3-L	S_Precentral-2-L	G_Angular-1-L
G_Cingulum_Post-2-L	G_Rolandic_Oper-1-L	S_Intraparietal-2-L	S_Precentral-3-L	G_Angular-2-L
G_Cingulum_Post-2-R	G_Rolandic_Oper-2-L	S_Intraoccipital-1-L	S_Precentral-4-L	G_Angular-3-L
G_Precuneus-2-L	G_Temporal_Sup-2-L	G_Occipital_Mid-1-L	S_Precentral-5-L	G_Parietal_Inf-1-L
G_Precuneus-2-R	S_Sup_Temporal-2-L	G_Occipital_Mid-2-L	S_Precentral-6-L	G_Occipital_Mid-1-L
G_Precuneus-4-L	S_Sup_Temporal-3-L	G_Occipital_Mid-3-L	S_Rolando-1-L	G_Occipital_Mid-2-L
G_Precuneus-7-R	G_Temporal_Pole_Sup-1-L	G_Temporal_Sup-1-R	S_Rolando-2-L	G_Occipital_Mid-3-L
G_Cuneus-1-R	G_Temporal_Pole_Sup-2-L	G_Temporal_Sup-2-R	S_Rolando-3-L	G_Occipital_Mid-4-L
G_Lingual-5-R	G_Temporal_Pole_Mid-1-L	G_Temporal_Mid-4-L	S_Rolando-4-L	G_Insula-posterior-1-L
G_ParaHippocampal-2-R	G_Temporal_Pole_Mid-2-L	G_Temporal_Inf-5-L	G_Parietal_Sup-1-L	G_Rolandic_Oper-2-L
G_Fusiform-1-R	G_Temporal_Pole_Mid-3-L	G_Supp_Motor_Area-2-L	G_Rolandic_Oper-1-L	G_Temporal_Sup-3-L
G_Fusiform-2-R	G_Hippocampus-1-L	G_Supp_Motor_Area-2-R	G_Rolandic_Oper-2-L	G_Temporal_Sup-4-L
G_Fusiform-3-R	N_Amygdala-1-L	G_Supp_Motor_Area-3-R	G_Frontal_Med_Orb-1-L	S_Sup_Temporal-2-L
G_Fusiform-4-R	N_Caudate-5-L	G_Paracentral_Lobule-4-R	G_Supp_Motor_Area-2-L	S_Sup_Temporal-3-L
G_Fusiform-5-R	N_Caudate-6-L	G_Fusiform-7-L	G_Supp_Motor_Area-3-L	S_Sup_Temporal-4-L
G_Fusiform-6-R	N_Pallidum-1-L	N_Caudate-6-R	N_Caudate-1-R	S_Sup_Temporal-5-L
G_Fusiform-7-R	N_Putamen-2-L	N_Caudate-7-R	N_Caudate-2-R	G_Temporal_Mid-1-L
N_Amygdala-1-R	N_Putamen-3-L	N_Thalamus-3-R	N_Caudate-4-L	G_Temporal_Mid-2-L
N_Pallidum-1-R	N_Thalamus-2-L	N_Thalamus-4-R	N_Caudate-4-R	G_Temporal_Mid-3-L
N_Putamen-2-R	N_Thalamus-3-L	N_Thalamus-6-R	N_Caudate-5-L	G_Temporal_Mid-4-L
N_Putamen-3-R	N_Thalamus-5-L	N_Thalamus-7-R	N_Caudate-6-L	G_Temporal_Inf-5-L

Supplementary Table 4. Component Loadings of the Mean Diffusivity (MD) Partial Least Squares Model for Language Outcomes.

pALF				
**Component 1**	**Component 2**	**Component 3**	**Component 4**	**Component 5**
G_Occipital_Pole-1-L	G_Frontal_Sup_Orb-1-L	S_Precentral-2-L	S_Sup_Frontal-1-L	G_Frontal_Sup-3-R
G_Occipital_Lat-1-L	G_Frontal_Sup_Orb-1-R	S_Precentral-3-L	S_Inf_Frontal-1-L	S_Sup_Frontal-4-R
G_Occipital_Lat-2-L	G_Frontal_Inf_Orb-1-L	S_Precentral-3-R	S_Inf_Frontal-2-L	S_Sup_Frontal-5-R
G_Occipital_Lat-3-L	G_Frontal_Inf_Orb-2-L	S_Precentral-6-L	G_Frontal_Inf_Tri-1-L	G_Frontal_Mid-1-R
G_Occipital_Lat-4-L	S_Olfactory-1-L	S_Precentral-6-R	G_Frontal_Mid_Orb-1-L	G_Frontal_Mid-2-R
G_Occipital_Lat-4-R	S_Olfactory-1-R	S_Rolando-2-R	G_Frontal_Mid_Orb-2-L	G_Supramarginal-4-L
G_Occipital_Lat-5-L	G_Insula-anterior-5-L	S_Rolando-3-R	G_Frontal_Inf_Orb-1-L	G_Supramarginal-4-R
G_Occipital_Sup-2-L	G_Insula-posterior-1-L	S_Rolando-4-L	G_Frontal_Inf_Orb-1-R	G_SupraMarginal-5-L
G_Occipital_Mid-1-L	G_Rolandic_Oper-1-L	S_Rolando-4-R	S_Orbital-1-L	G_SupraMarginal-6-L
G_Occipital_Mid-2-L	G_Rolandic_Oper-2-L	G_Parietal_Sup-1-R	S_Orbital-2-L	G_SupraMarginal-7-L
G_Occipital_Inf-1-L	G_Temporal_Sup-1-L	G_Parietal_Sup-2-L	S_Precentral-1-L	G_Angular-1-L
G_Occipital_Inf-2-L	G_Temporal_Sup-2-L	G_Parietal_Sup-2-R	S_Precentral-4-L	G_Angular-1-R
G_Temporal_Inf-4-L	G_Temporal_Sup-3-L	G_Supp_Motor_Area-3-L	S_Precentral-5-L	G_Angular-2-L
S_Parietooccipital-5-L	G_Temporal_Sup-4-L	G_Supp_Motor_Area-3-R	S_Rolando-1-L	G_Angular-3-L
S_Parietooccipital-6-L	S_Sup_Temporal-1-L	S_Cingulate-3-R	S_Rolando-1-R	G_Angular-3-R
S_Parietooccipital-6-R	S_Sup_Temporal-2-L	S_Cingulate-4-L	S_Rolando-2-L	G_Occipital_Pole-1-L
G_Cuneus-1-L	G_Temporal_Mid-1-L	S_Cingulate-4-R	S_Postcentral-1-R	G_Occipital_Pole-1-R
G_Cuneus-1-R	G_Temporal_Mid-2-L	S_Cingulate-7-R	G_Occipital_Pole-1-L	G_Occipital_Lat-1-R
G_Cuneus-2-L	G_Temporal_Mid-3-L	G_Paracentral_Lobule-2-L	G_Occipital_Mid-1-R	G_Occipital_Lat-3-R
G_Cuneus-2-R	G_Temporal_Inf-1-L	G_Paracentral_Lobule-2-R	G_Insula-anterior-2-L	G_Occipital_Mid-3-L
G_Calcarine-1-L	G_Temporal_Inf-2-L	G_Paracentral_Lobule-3-L	G_Insula-anterior-4-L	G_Occipital_Mid-4-L
G_Calcarine-2-L	G_Temporal_Pole_Sup-1-L	G_Paracentral_Lobule-3-R	G_Rolandic_Oper-1-L	S_Sup_Temporal-3-L
G_Calcarine-2-R	G_Temporal_Pole_Sup-2-L	G_Paracentral_Lobule-4-L	G_Temporal_Sup-1-L	S_Sup_Temporal-4-L
G_Calcarine-3-L	G_subcallosal-1-L	G_Paracentral_Lobule-4-R	G_Temporal_Sup-3-R	S_Sup_Temporal-5-L
G_Lingual-3-L	G_subcallosal-1-R	S_Parietooccipital-6-L	S_Sup_Temporal-1-L	G_Temporal_Mid-3-L
G_Lingual-4-L	N_Amygdala-1-L	G_Cuneus-1-L	S_Sup_Temporal-1-R	G_Temporal_Mid-4-L
G_Lingual-5-L	N_Caudate-3-L	G_Calcarine-1-L	G_Temporal_Mid-3-R	G_Frontal_Sup_Medial-1-R
G_Lingual-6-L	N_Putamen-2-L	G_Calcarine-2-L	G_Cingulum_Mid-1-L	G_Frontal_Sup_Medial-2-R
G_Fusiform-7-L	N_Putamen-3-L	G_Lingual-3-L	N_Caudate-2-L	G_Supp_Motor_Area-2-R

Supplementary Table 5. Component Loadings of the pALF Partial Least Squares Model for Language Outcomes.

Task fMRI				
**Component 1**	**Component 2**	**Component 3**	**Component 4**	**Component 5**
S_Sup_Frontal-1-L	G_Parietal_Sup-3-L	G_Hippocampus-2-L	S_Precentral-5-L	G_Occipital_Pole-1-L
G_Frontal_Inf_Orb-1-L	G_Parietal_Sup-3-R	G_Hippocampus-2-R	S_Rolando-1-L	G_Occipital_Lat-1-L
G_Frontal_Inf_Orb-1-R	G_Parietal_Inf-1-L	N_Amygdala-1-L	G_Parietal_Sup-3-L	G_Occipital_Lat-3-L
G_Frontal_Inf_Orb-2-L	G_Parietal_Inf-1-R	N_Caudate-4-L	G_Parietal_Sup-5-L	G_Calcarine-3-L
S_Orbital-1-L	S_Intraparietal-2-R	N_Caudate-5-L	G_SupraMarginal-2-L	G_Fusiform-1-L
S_Orbital-1-R	G_Occipital_Pole-1-L	N_Caudate-6-L	G_Supramarginal-4-L	G_Fusiform-2-L
S_Olfactory-1-L	G_Occipital_Pole-1-R	N_Caudate-6-R	G_SupraMarginal-5-L	G_Fusiform-2-R
S_Olfactory-1-R	G_Occipital_Lat-1-L	N_Caudate-7-L	G_Angular-2-L	G_Fusiform-3-L
S_Precentral-3-R	G_Occipital_Lat-1-R	N_Caudate-7-R	G_Angular-2-R	G_Fusiform-3-R
G_SupraMarginal-7-R	G_Occipital_Lat-2-L	N_Putamen-2-L	G_Angular-3-R	G_Fusiform-7-L
G_Angular-1-L	G_Occipital_Lat-2-R	N_Putamen-2-R	S_Intraoccipital-1-L	G_Fusiform-7-R
G_Angular-1-R	G_Occipital_Lat-3-L	N_Putamen-3-L	S_Intraoccipital-1-R	N_Caudate-1-L
G_Angular-2-L	G_Occipital_Lat-3-R	N_Putamen-3-R	G_Occipital_Sup-1-L	N_Caudate-1-R
G_Occipital_Pole-1-L	G_Occipital_Sup-1-R	N_Thalamus-1-L	G_Occipital_Sup-1-R	N_Caudate-3-L
G_Insula-anterior-2-L	G_Occipital_Sup-2-R	N_Thalamus-1-R	G_Occipital_Mid-4-L	N_Caudate-3-R
S_Sup_Temporal-1-R	G_Paracentral_Lobule-3-L	N_Thalamus-2-L	G_Occipital_Mid-4-R	N_Caudate-4-R
G_Temporal_Mid-1-R	G_Paracentral_Lobule-4-L	N_Thalamus-3-L	G_Precuneus-3-R	N_Caudate-5-R
G_Temporal_Mid-2-R	G_Paracentral_Lobule-4-R	N_Thalamus-3-R	G_Precuneus-6-L	N_Pallidum-1-L
G_Temporal_Inf-2-L	G_Precuneus-6-L	N_Thalamus-4-L	G_Precuneus-7-L	N_Pallidum-1-R
G_Temporal_Inf-2-R	G_Precuneus-6-R	N_Thalamus-5-L	G_Precuneus-7-R	N_Putamen-3-L
G_Temporal_Inf-3-R	G_Precuneus-8-L	N_Thalamus-5-R	G_Precuneus-8-L	N_Thalamus-1-L
G_Temporal_Pole_Mid-1-R	G_Precuneus-8-R	N_Thalamus-6-L	G_Precuneus-8-R	N_Thalamus-1-R
G_Temporal_Pole_Mid-2-L	G_Precuneus-9-R	N_Thalamus-6-R	S_Parietooccipital-2-L	N_Thalamus-2-R
S_Anterior_Rostral-1-R	G_Calcarine-3-L	N_Thalamus-7-L	S_Parietooccipital-2-R	N_Thalamus-3-R
G_Frontal_Med_Orb-2-R	G_Calcarine-3-R	N_Thalamus-7-R	S_Parietooccipital-3-L	N_Thalamus-5-R
G_subcallosal-1-L	G_Lingual-5-L	N_Thalamus-8-L	S_Parietooccipital-3-R	N_Thalamus-6-R
G_subcallosal-1-R	G_Lingual-5-R	N_Thalamus-8-R	S_Parietooccipital-6-L	N_Thalamus-7-R
G_Supp_Motor_Area-2-R	G_Fusiform-7-L	N_Thalamus-9-L	S_Parietooccipital-6-R	N_Thalamus-9-L
G_Cingulum_Ant-1-R	G_Fusiform-7-R	N_Thalamus-9-R	G_Cuneus-1-R	N_Thalamus-9-R

Supplementary Table 6. Component Loadings of the functional Magnetic Resonance Imaging (fMRI) Partial Least Squares Model for Language Outcomes.

Vbm GM				
**Component 1**	**Component 2**	**Component 3**	**Component 4**	**Component 5**
G_Temporal_Sup-4-L	S_Sup_Frontal-6-L	G_Frontal_Sup-3-L	G_Frontal_Sup-3-L	G_Frontal_Sup-3-L
G_Temporal_Pole_Sup-1-L	S_Precentral-2-L	G_Frontal_Mid-3-L	S_Sup_Frontal-6-L	S_Sup_Frontal-4-L
G_Temporal_Pole_Sup-1-R	S_Precentral-3-L	G_Frontal_Mid-4-L	G_Frontal_Mid-4-L	G_Frontal_Inf_Tri-1-L
G_Temporal_Pole_Sup-2-L	S_Rolando-4-L	G_Frontal_Mid-5-L	G_Frontal_Mid-5-L	S_Precentral-1-L
G_Temporal_Pole_Mid-2-L	S_Postcentral-2-L	S_Inf_Frontal-2-L	S_Precentral-1-L	S_Precentral-2-L
G_Temporal_Pole_Mid-2-R	S_Postcentral-3-L	G_Frontal_Inf_Tri-1-L	S_Precentral-2-L	S_Precentral-5-L
G_Temporal_Pole_Mid-3-L	G_Parietal_Sup-1-L	S_Precentral-1-L	S_Precentral-3-L	S_Postcentral-1-L
G_Supp_Motor_Area-2-R	G_Parietal_Sup-2-L	S_Precentral-2-L	S_Precentral-4-L	G_Parietal_Sup-1-L
G_Cingulum_Post-3-R	G_Parietal_Sup-4-L	S_Precentral-4-L	S_Precentral-6-L	G_Parietal_Sup-2-L
G_ParaHippocampal-2-R	G_Supramarginal-1-L	S_Precentral-5-L	S_Rolando-2-L	G_Supramarginal-1-L
N_Caudate-1-L	G_SupraMarginal-2-L	S_Precentral-6-L	S_Rolando-3-L	G_SupraMarginal-2-L
N_Caudate-2-L	G_Supramarginal-3-L	G_SupraMarginal-6-L	S_Rolando-4-L	G_SupraMarginal-5-L
N_Caudate-3-L	G_Supramarginal-4-L	G_Insula-anterior-3-L	S_Postcentral-1-L	G_SupraMarginal-7-L
N_Caudate-4-L	G_SupraMarginal-5-L	G_Insula-anterior-4-L	G_Parietal_Sup-1-L	S_Intraoccipital-1-L
N_Caudate-5-L	G_SupraMarginal-6-L	G_Frontal_Sup_Medial-3-L	G_Parietal_Sup-2-L	G_Insula-anterior-2-L
N_Caudate-6-L	G_SupraMarginal-7-L	G_Supp_Motor_Area-1-L	G_Parietal_Sup-4-L	G_Insula-anterior-3-L
N_Caudate-7-L	G_Angular-1-L	G_Supp_Motor_Area-2-L	S_Intraparietal-1-L	G_Insula-anterior-4-L
N_Pallidum-1-L	G_Angular-2-L	G_Supp_Motor_Area-3-L	G_Insula-anterior-4-L	G_Insula-anterior-5-L
N_Pallidum-1-R	S_Intraparietal-1-L	S_Cingulate-1-L	G_Supp_Motor_Area-3-L	G_Insula-posterior-1-L
N_Putamen-2-R	S_Intraparietal-2-L	N_Caudate-3-L	S_Cingulate-7-L	G_Rolandic_Oper-1-L
N_Putamen-3-R	S_Intraparietal-3-L	N_Caudate-4-L	N_Caudate-1-L	G_Rolandic_Oper-2-L
N_Thalamus-2-L	G_Occipital_Mid-3-L	N_Caudate-5-L	N_Caudate-4-L	G_Temporal_Sup-1-L
N_Thalamus-2-R	S_Sup_Temporal-3-L	N_Caudate-6-L	N_Caudate-5-L	G_Temporal_Sup-2-L
N_Thalamus-5-L	S_Sup_Temporal-4-L	N_Putamen-2-L	N_Caudate-6-L	G_Supp_Motor_Area-2-L
N_Thalamus-5-R	S_Sup_Temporal-5-L	N_Thalamus-3-L	N_Caudate-7-L	S_Cingulate-7-L
N_Thalamus-6-L	G_Temporal_Mid-3-L	N_Thalamus-3-R	N_Pallidum-1-L	N_Pallidum-1-L
N_Thalamus-7-L	G_Temporal_Mid-4-L	N_Thalamus-4-L	N_Putamen-2-L	N_Putamen-2-L
N_Thalamus-8-L	G_Temporal_Inf-5-L	N_Thalamus-4-R	N_Putamen-3-L	N_Putamen-3-L
N_Thalamus-8-R	N_Pallidum-1-L	N_Thalamus-7-R	N_Thalamus-5-L	N_Thalamus-4-L

Supplementary Table 7. Component Loadings of the Voxel-Based Morphometry of Gray Matter (Vbm GM) Partial Least Squares Model for Language Outcomes.

Vbm WM				
**Component 1**	**Component 2**	**Component 3**	**Component 4**	**Component 5**
G_Occipital_Pole-1-R	S_Precentral-2-L	G_Frontal_Sup-1-R	G_Frontal_Mid-1-L	G_Frontal_Mid-5-L
G_Insula-anterior-1-L	S_Postcentral-3-R	G_Frontal_Mid-4-L	G_Frontal_Mid-4-L	G_Frontal_Inf_Tri-1-L
G_Insula-anterior-1-R	G_Parietal_Sup-1-R	S_Orbital-1-L	G_Frontal_Mid-5-L	S_Precentral-1-L
G_Temporal_Sup-1-L	G_Parietal_Sup-4-L	S_Precentral-6-L	S_Inf_Frontal-2-L	G_SupraMarginal-7-L
G_Temporal_Sup-1-R	G_SupraMarginal-2-L	S_Precentral-6-R	G_Frontal_Inf_Tri-1-L	G_Rolandic_Oper-1-L
G_Temporal_Sup-2-R	G_SupraMarginal-6-L	S_Postcentral-1-L	S_Precentral-1-L	G_Rolandic_Oper-2-L
S_Sup_Temporal-1-L	S_Intraparietal-1-L	S_Postcentral-3-L	S_Precentral-2-L	G_Temporal_Sup-3-L
S_Sup_Temporal-1-R	S_Intraparietal-1-R	G_Parietal_Sup-1-L	S_Precentral-4-L	G_Temporal_Sup-4-L
G_Temporal_Mid-1-R	S_Intraoccipital-1-L	G_Parietal_Sup-3-L	S_Precentral-5-L	S_Sup_Temporal-2-L
G_Temporal_Pole_Sup-1-R	S_Intraoccipital-1-R	G_Parietal_Sup-4-L	S_Rolando-1-L	S_Sup_Temporal-4-L
G_Temporal_Pole_Sup-2-L	S_Cingulate-3-L	G_SupraMarginal-6-R	S_Rolando-3-L	S_Sup_Temporal-5-L
G_Temporal_Pole_Mid-3-L	S_Cingulate-5-L	G_Parietal_Inf-1-R	S_Rolando-4-L	G_Temporal_Mid-1-L
G_Temporal_Pole_Mid-3-R	G_Cingulum_Mid-2-L	G_Occipital_Sup-1-R	S_Postcentral-1-L	G_Temporal_Mid-2-L
G_ParaHippocampal-1-L	G_Cingulum_Mid-2-R	G_Temporal_Inf-5-R	S_Postcentral-3-L	G_Temporal_Mid-3-L
G_ParaHippocampal-1-R	G_Cingulum_Mid-3-L	G_Temporal_Pole_Mid-1-L	G_Parietal_Sup-1-L	G_Temporal_Mid-4-L
G_ParaHippocampal-3-L	G_Cingulum_Mid-3-R	G_Temporal_Pole_Mid-2-L	G_Parietal_Sup-4-R	G_Temporal_Inf-4-L
G_ParaHippocampal-3-R	G_Cingulum_Post-3-R	G_Temporal_Pole_Mid-2-R	S_Intraoccipital-1-R	G_Temporal_Inf-5-L
G_ParaHippocampal-4-L	G_Paracentral_Lobule-1-L	G_Precuneus-8-L	G_Rolandic_Oper-1-L	G_Temporal_Pole_Mid-2-L
G_ParaHippocampal-4-R	S_Parietooccipital-5-L	G_Precuneus-8-R	G_Rolandic_Oper-2-L	S_Cingulate-3-L
N_Caudate-1-L	S_Parietooccipital-5-R	G_Calcarine-1-R	G_Supp_Motor_Area-2-L	S_Cingulate-5-L
N_Caudate-1-R	S_Parietooccipital-6-L	G_Calcarine-2-L	G_Cingulum_Mid-2-L	S_Cingulate-5-R
N_Caudate-4-R	G_Lingual-2-L	N_Caudate-1-R	G_Cingulum_Mid-2-R	S_Cingulate-6-L
N_Thalamus-3-R	G_Lingual-3-R	N_Caudate-2-R	G_Paracentral_Lobule-2-L	G_Cingulum_Mid-1-L
N_Thalamus-4-R	G_Lingual-4-L	N_Caudate-4-R	N_Amygdala-1-L	G_Cingulum_Mid-1-R
N_Thalamus-6-L	G_Lingual-5-R	N_Caudate-7-R	N_Pallidum-1-L	G_Cingulum_Mid-3-R
N_Thalamus-7-L	G_Lingual-6-L	N_Putamen-2-L	N_Putamen-3-L	G_Precuneus-3-L
N_Thalamus-7-R	G_Lingual-6-R	N_Thalamus-1-R	N_Thalamus-2-L	S_Parietooccipital-5-L
N_Thalamus-8-L	G_Fusiform-7-R	N_Thalamus-2-R	N_Thalamus-3-L	S_Parietooccipital-6-L
N_Thalamus-8-R	N_Putamen-2-L	N_Thalamus-5-L	N_Thalamus-5-L	N_Caudate-7-L

Supplementary Table 8. Component Loadings of the Voxel-Based Morphometry of White Matter (Vbm WM) Partial Least Squares Model for Language Outcomes.

rsfMRI				
**Component 1**	**Component 2**	**Component 3**	**Component 4**	**Component 5**
G_Occipital_Lat-2-R and S_Rolando-2-R	S_Sup_Frontal-6-L and G_Frontal_Sup-2-L	G_Angular-1-R and G_Frontal_Inf_Orb-1-R	S_Sup_Temporal-3-L and G_SupraMarginal-7-L	G_Frontal_Mid-3-L and S_Sup_Frontal-5-R
G_Occipital_Lat-4-R and S_Rolando-2-R	S_Precentral-2-L and G_Frontal_Sup-2-L	G_Temporal_Inf-4-R and G_SupraMarginal-2-L	G_Occipital_Mid-1-R and G_Angular-3-L	G_Frontal_Mid-3-L and S_Sup_Frontal-6-R
G_Occipital_Lat-5-R and S_Rolando-2-R	S_Sup_Frontal-5-L and G_Frontal_Sup-2-R	G_Temporal_Mid-2-R and G_Angular-1-L	G_Occipital_Mid-2-R and G_Angular-3-L	S_Sup_Frontal-5-R and G_Frontal_Mid-3-L
G_Occipital_Sup-1-L and G_Occipital_Lat-1-L	S_Sup_Frontal-6-L and G_Frontal_Sup-2-R	G_Frontal_Inf_Orb-1-R and G_Angular-1-R	G_Temporal_Inf-5-L and G_Occipital_Lat-5-R	S_Sup_Frontal-6-R and G_Frontal_Mid-3-L
G_Cuneus-1-L and G_Occipital_Lat-1-L	S_Cingulate-2-R and S_Sup_Frontal-4-L	G_Temporal_Inf-2-L and G_Angular-2-L	G_Angular-3-L and G_Occipital_Mid-1-R	G_Precuneus-6-R and G_Frontal_Mid-3-L
S_Cingulate-2-R and G_Occipital_Lat-1-R	S_Cingulate-3-R and S_Sup_Frontal-4-L	G_Temporal_Mid-2-R and G_Angular-2-R	G_Angular-3-L and G_Occipital_Mid-2-R	G_Supramarginal-1-R and S_Precentral-6-L
G_Occipital_Sup-1-L and G_Occipital_Lat-2-L	S_Sup_Frontal-6-L and S_Sup_Frontal-4-R	G_Temporal_Inf-2-L and G_Angular-2-R	G_Occipital_Mid-3-L and G_Occipital_Mid-2-R	S_Postcentral-1-L and S_Precentral-6-R
G_Occipital_Sup-1-R and G_Occipital_Lat-2-L	G_Frontal_Sup-2-R and S_Sup_Frontal-5-L	G_Temporal_Inf-2-R and G_Angular-2-R	G_Temporal_Inf-5-L and G_Occipital_Mid-2-R	S_Postcentral-3-L and S_Precentral-6-R
S_Parietooccipital-3-L and G_Occipital_Lat-2-L	G_Supp_Motor_Area-2-R and S_Sup_Frontal-5-L	G_ParaHippocampal-2-L and G_Occipital_Mid-4-L	G_Occipital_Mid-2-R and G_Occipital_Mid-3-L	S_Rolando-4-R and S_Rolando-1-L
S_Parietooccipital-3-R and G_Occipital_Lat-2-L	S_Sup_Frontal-6-L and S_Sup_Frontal-5-R	G_ParaHippocampal-4-L and G_Occipital_Mid-4-L	G_Occipital_Mid-3-R and G_Occipital_Mid-3-L	G_Cuneus-2-R and S_Rolando-2-L
S_Parietooccipital-6-L and G_Occipital_Lat-2-L	S_Cingulate-4-L and S_Sup_Frontal-5-R	G_ParaHippocampal-2-L and G_Occipital_Mid-4-R	S_Sup_Temporal-4-R and G_Occipital_Mid-3-L	S_Postcentral-1-L and S_Rolando-2-R
S_Parietooccipital-6-R and G_Occipital_Lat-2-L	G_Frontal_Sup-2-L and S_Sup_Frontal-6-L	G_Cingulum_Mid-3-R and G_Insula-anterior-2-R	G_Occipital_Mid-3-L and G_Occipital_Mid-3-R	S_Postcentral-1-L and S_Rolando-3-R
G_Cuneus-1-L and G_Occipital_Lat-2-L	G_Frontal_Sup-2-R and S_Sup_Frontal-6-L	S_Cingulate-6-R and G_Insula-anterior-4-R	S_Sup_Temporal-4-L and G_Occipital_Mid-3-R	S_Rolando-1-L and S_Rolando-4-R
S_Rolando-2-R and G_Occipital_Lat-2-R	S_Sup_Frontal-4-R and S_Sup_Frontal-6-L	S_Cingulate-6-R and G_Temporal_Sup-2-L	G_Temporal_Mid-4-L and G_Occipital_Mid-3-R	S_Precentral-6-R and S_Postcentral-1-L
S_Cingulate-2-R and G_Occipital_Lat-2-R	S_Sup_Frontal-5-R and S_Sup_Frontal-6-L	S_Cingulate-5-R and G_Temporal_Sup-3-L		S_Rolando-2-R and S_Postcentral-1-L
S_Cingulate-3-R and G_Occipital_Lat-2-R	G_Supp_Motor_Area-2-R and S_Sup_Frontal-6-L	G_Precuneus-3-R and G_Temporal_Mid-1-R		S_Rolando-3-R and S_Postcentral-1-L
S_Parietooccipital-6-L and G_Occipital_Lat-2-R	S_Cingulate-2-R and G_Frontal_Mid-4-L	G_Precuneus-7-R and G_Temporal_Mid-1-R		S_Postcentral-2-R and S_Postcentral-1-L

G_Cuneus-1-R and G_Occipital_Lat-2-R<br>S_Parietooccipital-6-L and G_Occipital_Lat-3-R<br>S_Rolando-2-R and G_Occipital_Lat-4-R<br>S_Cingulate-2-R and G_Occipital_Lat-4-R<br>S_Cingulate-3-R and G_Occipital_Lat-4-R<br>S_Parietooccipital-3-R and G_Occipital_Lat-4-R<br>S_Rolando-2-R and G_Occipital_Lat-5-R<br>S_Cingulate-2-R and G_Occipital_Lat-5-R<br>G_Occipital_Lat-1-L and G_Occipital_Sup-1-L<br>G_Occipital_Lat-2-L and G_Occipital_Sup-1-L<br>G_Occipital_Lat-2-L and G_Occipital_Sup-1-R<br>S_Cingulate-5-R and G_Temporal_Inf-5-R<br>S_Cingulate-6-R and G_Temporal_Inf-5-R<br>G_Occipital_Lat-1-R and S_Cingulate-2-R<br>G_Occipital_Lat-2-R and S_Cingulate-2-R<br>G_Occipital_Lat-4-R and S_Cingulate-2-R<br>G_Occipital_Lat-5-R and S_Cingulate-2-R<br>G_Occipital_Lat-2-R and S_Cingulate-3-R	S_Cingulate-2-R and G_Frontal_Mid-5-L<br>G_Frontal_Sup-2-L and S_Precentral-2-L<br>S_Anterior_Rostral-1-L and G_Insula-anterior-3-R<br>G_Frontal_Med_Orb-2-L and G_Insula-anterior-3-R<br>G_Frontal_Med_Orb-2-R and G_Insula-anterior-3-R<br>G_Frontal_Med_Orb-2-L and G_Insula-anterior-4-L<br>G_Frontal_Med_Orb-2-R and G_Insula-anterior-4-L<br>S_Anterior_Rostral-1-L and G_Insula-anterior-4-R<br>S_Anterior_Rostral-1-R and G_Insula-anterior-4-R<br>G_Frontal_Med_Orb-2-L and G_Insula-anterior-4-R<br>G_Frontal_Med_Orb-2-R and G_Insula-anterior-4-R<br>G_Insula-anterior-3-R and S_Anterior_Rostral-1-L	G_Precuneus-3-R and G_Temporal_Mid-2-L<br>G_Angular-1-L and G_Temporal_Mid-2-R<br>G_Angular-2-R and G_Temporal_Mid-2-R<br>G_Cingulum_Post-2-R and G_Temporal_Mid-2-R<br>G_Precuneus-3-R and G_Temporal_Mid-2-R<br>G_Precuneus-7-L and G_Temporal_Mid-2-R<br>G_Precuneus-7-R and G_Temporal_Mid-2-R<br>G_Precuneus-7-R and G_Temporal_Mid-3-R<br>G_Angular-2-L and G_Temporal_Inf-2-L<br>G_Angular-2-R and G_Temporal_Inf-2-L<br>G_Precuneus-3-R and G_Temporal_Inf-2-L<br>G_Precuneus-7-L and G_Temporal_Inf-2-L<br>G_Angular-2-R and G_Temporal_Inf-2-R<br>G_SupraMarginal-2-L and G_Temporal_Inf-4-R<br>G_Temporal_Sup-3-L and S_Cingulate-5-R<br>G_Insula-anterior-4-R and S_Cingulate-6-R<br>G_Temporal_Sup-2-L and S_Cingulate-6-R<br>G_Insula-anterior-2-R and G_Cingulum_Mid-3-R	G_Temporal_Inf-5-L and G_Occipital_Mid-3-R<br>G_Temporal_Inf-5-L and G_Occipital_Inf-2-R<br>G_Temporal_Sup-4-R and G_Temporal_Sup-4-L<br>S_Sup_Temporal-3-R and G_Temporal_Sup-4-L<br>G_Temporal_Mid-3-R and G_Temporal_Sup-4-L<br>G_Temporal_Sup-4-L and G_Temporal_Sup-4-R<br>G_SupraMarginal-7-L and S_Sup_Temporal-3-L<br>S_Sup_Temporal-3-R and S_Sup_Temporal-3-L<br>S_Sup_Temporal-5-L and S_Sup_Temporal-3-L<br>G_Temporal_Mid-3-R and S_Sup_Temporal-3-L<br>G_Temporal_Sup-4-L and S_Sup_Temporal-3-R<br>S_Sup_Temporal-3-L and S_Sup_Temporal-3-R<br>G_Occipital_Mid-3-R and S_Sup_Temporal-4-L<br>S_Sup_Temporal-4-R and S_Sup_Temporal-4-L<br>S_Sup_Temporal-5-R and S_Sup_Temporal-4-L	G_Parietal_Sup-1-R and S_Postcentral-1-L<br>G_Parietal_Sup-3-R and S_Postcentral-1-L<br>S_Postcentral-2-L and S_Postcentral-1-R<br>S_Postcentral-1-R and S_Postcentral-2-L<br>G_Supramarginal-1-R and S_Postcentral-2-L<br>S_Postcentral-1-L and S_Postcentral-2-R<br>S_Postcentral-3-L and S_Postcentral-2-R<br>S_Precentral-6-R and S_Postcentral-3-L<br>S_Postcentral-2-R and S_Postcentral-3-L<br>G_Cuneus-2-R and S_Postcentral-3-L<br>G_Calcarine-1-R and S_Postcentral-3-L<br>G_Calcarine-2-L and S_Postcentral-3-L<br>G_Calcarine-2-R and S_Postcentral-3-L<br>G_Lingual-6-R and S_Postcentral-3-L<br>S_Postcentral-1-L and G_Parietal_Sup-1-R<br>G_Supramarginal-1-R and G_Parietal_Sup-3-L<br>S_Postcentral-1-L and G_Parietal_Sup-3-R<br>G_Supramarginal-3-L and G_Parietal_Sup-3-R

G_Occipital_Lat-4-R and S_Cingulate-3-R<br>G_Temporal_Inf-5-R and S_Cingulate-5-R<br>G_Temporal_Inf-5-R and S_Cingulate-6-R<br>G_Occipital_Lat-2-L and S_Parietooccipital-3-L<br>G_Occipital_Lat-2-L and S_Parietooccipital-3-R<br>G_Occipital_Lat-4-R and S_Parietooccipital-3-R<br>G_Occipital_Lat-2-L and S_Parietooccipital-6-L<br>G_Occipital_Lat-2-R and S_Parietooccipital-6-L<br>G_Occipital_Lat-3-R and S_Parietooccipital-6-L<br>G_Occipital_Lat-2-L and S_Parietooccipital-6-R<br>G_Occipital_Lat-1-L and G_Cuneus-1-L<br>G_Occipital_Lat-2-L and G_Cuneus-1-L<br>G_Occipital_Lat-2-R and G_Cuneus-1-R	G_Insula-anterior-4-R and S_Anterior_Rostral-1-L<br>G_Insula-anterior-4-R and S_Anterior_Rostral-1-R<br>G_Insula-anterior-3-R and G_Frontal_Med_Orb-2-L<br>G_Insula-anterior-4-L and G_Frontal_Med_Orb-2-L<br>G_Insula-anterior-4-R and G_Frontal_Med_Orb-2-L<br>G_Insula-anterior-3-R and G_Frontal_Med_Orb-2-R<br>G_Insula-anterior-4-L and G_Frontal_Med_Orb-2-R<br>G_Insula-anterior-4-R and G_Frontal_Med_Orb-2-R<br>S_Sup_Frontal-5-L and G_Supp_Motor_Area-2-R<br>S_Sup_Frontal-6-L and G_Supp_Motor_Area-2-R<br>S_Sup_Frontal-4-L and S_Cingulate-2-R<br>G_Frontal_Mid-4-L and S_Cingulate-2-R<br>G_Frontal_Mid-5-L and S_Cingulate-2-R	G_Temporal_Mid-2-R and G_Cingulum_Post-2-R<br>G_Temporal_Mid-1-R and G_Precuneus-3-R<br>G_Temporal_Mid-2-L and G_Precuneus-3-R<br>G_Temporal_Mid-2-R and G_Precuneus-3-R<br>G_Temporal_Inf-2-L and G_Precuneus-3-R<br>G_Temporal_Mid-2-R and G_Precuneus-7-L<br>G_Temporal_Inf-2-L and G_Precuneus-7-L<br>G_Temporal_Mid-1-R and G_Precuneus-7-R<br>G_Temporal_Mid-2-R and G_Precuneus-7-R<br>G_Temporal_Mid-3-R and G_Precuneus-7-R<br>G_Occipital_Mid-4-L and G_ParaHippocampal-2-L<br>G_Occipital_Mid-4-R and G_ParaHippocampal-2-L<br>G_Occipital_Mid-4-L and G_ParaHippocampal-4-L	G_Occipital_Mid-3-L and S_Sup_Temporal-4-R<br>S_Sup_Temporal-4-L and S_Sup_Temporal-4-R<br>S_Sup_Temporal-3-L and S_Sup_Temporal-5-L<br>G_Temporal_Mid-3-L and S_Sup_Temporal-5-L<br>S_Sup_Temporal-4-L and S_Sup_Temporal-5-R<br>S_Sup_Temporal-5-L and G_Temporal_Mid-3-L<br>G_Temporal_Sup-4-L and G_Temporal_Mid-3-R<br>S_Sup_Temporal-3-L and G_Temporal_Mid-3-R<br>G_Occipital_Mid-3-R and G_Temporal_Mid-4-L<br>G_Temporal_Mid-4-R and G_Temporal_Mid-4-L<br>G_Temporal_Mid-4-L and G_Temporal_Mid-4-R<br>G_Temporal_Inf-5-L and G_Temporal_Mid-4-R<br>G_Occipital_Lat-5-R and G_Temporal_Inf-5-L<br>G_Occipital_Mid-2-R and G_Temporal_Inf-5-L<br>G_Occipital_Mid-3-R and G_Temporal_Inf-5-L	G_Supramarginal-1-R and G_Parietal_Sup-5-L<br>S_Precentral-6-L and G_Supramarginal-1-R<br>S_Postcentral-2-L and G_Supramarginal-1-R<br>G_Parietal_Sup-3-L and G_Supramarginal-1-R<br>G_Parietal_Sup-5-L and G_Supramarginal-1-R<br>G_Parietal_Sup-3-R and G_Supramarginal-3-L<br>G_Frontal_Mid-3-L and G_Precuneus-6-R<br>S_Rolando-2-L and G_Cuneus-2-R<br>S_Postcentral-3-L and G_Cuneus-2-R<br>S_Postcentral-3-L and G_Calcarine-1-R<br>S_Postcentral-3-L and G_Calcarine-2-L<br>S_Postcentral-3-L and G_Calcarine-2-R<br>S_Postcentral-3-L and G_Lingual-6-R

	S_Sup_Frontal-4-L and S_Cingulate-3-R<br>S_Sup_Frontal-5-R and S_Cingulate-4-L<br>N_Thalamus-2-R and N_Caudate-4-L<br>N_Thalamus-2-L and N_Caudate-7-R<br>N_Caudate-7-R and N_Thalamus-2-L<br>N_Caudate-4-L and N_Thalamus-2-R		G_Occipital_Inf-2-R and G_Temporal_Inf-5-L<br>G_Temporal_Mid-4-R and G_Temporal_Inf-5-L<br>G_Temporal_Inf-5-R and G_Temporal_Inf-5-L<br>G_Temporal_Inf-5-L and G_Temporal_Inf-5-R	

Supplementary Table 9. Component Loadings of the Functional Connectivity (rsfMRI) Partial Least Squares Model for Language Outcomes.

DTI				
**Component 1**	**Component 2**	**Component 3**	**Component 4**	**Component 5**
G_Angular-2-R and S_Inf_Frontal-2-R<br>N_Putamen-3-R and S_Postcentral-2-R<br>S_Inf_Frontal-2-R and G_Angular-2-R<br>G_Insula-posterior-1-R and G_Insula-anterior-3-R<br>G_Insula-anterior-3-R and G_Insula-posterior-1-R<br>G_Rolandic_Oper-1-R and G_Insula-posterior-1-R<br>G_Rolandic_Oper-2-R and G_Insula-posterior-1-R<br>S_Sup_Temporal-3-R and G_Insula-posterior-1-R<br>S_Sup_Temporal-4-R and G_Insula-posterior-1-R<br>G_Insula-posterior-1-R and G_Rolandic_Oper-1-R<br>G_Rolandic_Oper-2-R and G_Rolandic_Oper-1-R<br>G_Insula-posterior-1-R and G_Rolandic_Oper-2-R<br>G_Rolandic_Oper-1-R and G_Rolandic_Oper-2-R<br>G_Temporal_Sup-3-R and G_Rolandic_Oper-2-R<br>S_Sup_Temporal-3-R and G_Rolandic_Oper-2-R<br>G_Rolandic_Oper-2-R and G_Temporal_Sup-3-R	N_Putamen-2-R and G_Frontal_Sup-2-R<br>N_Putamen-3-R and G_Frontal_Sup-2-R<br>S_Postcentral-2-R and S_Rolando-1-R<br>N_Putamen-3-R and S_Rolando-3-R<br>N_Putamen-3-R and S_Rolando-4-R<br>S_Rolando-1-R and S_Postcentral-2-R<br>N_Putamen-3-R and G_Insula-posterior-1-R<br>G_Precuneus-3-L and S_Cingulate-1-L<br>G_Precuneus-3-L and S_Cingulate-3-L<br>G_Precuneus-3-L and S_Cingulate-4-L<br>N_Thalamus-5-R and S_Cingulate-4-R<br>G_Precuneus-3-L and S_Cingulate-5-L<br>N_Putamen-3-R and G_Paracentral_Lobule-2-R<br>N_Thalamus-5-R and G_Paracentral_Lobule-2-R<br>S_Cingulate-1-L and G_Precuneus-3-L	N_Putamen-2-L and G_Frontal_Inf_Tri-1-L<br>N_Putamen-2-R and G_Frontal_Inf_Tri-1-R<br>N_Putamen-3-L and S_Rolando-3-L<br>G_Insula-posterior-1-L and S_Rolando-4-L<br>N_Putamen-3-L and S_Rolando-4-L<br>N_Thalamus-5-L and S_Rolando-4-L<br>N_Thalamus-5-R and S_Rolando-4-R<br>N_Putamen-2-R and G_Insula-anterior-3-R<br>S_Rolando-4-L and G_Insula-posterior-1-L<br>N_Putamen-2-L and G_Supp_Motor_Area-3-L<br>N_Thalamus-5-L and G_Supp_Motor_Area-3-L<br>N_Thalamus-5-L and S_Cingulate-4-L<br>N_Thalamus-5-L and G_Paracentral_Lobule-2-L<br>G_Frontal_Inf_Tri-1-L and N_Putamen-2-L<br>G_Supp_Motor_Area-3-L and N_Putamen-2-L<br>N_Thalamus-5-L and N_Putamen-2-L	N_Putamen-2-L and G_Frontal_Sup_Orb-1-L<br>S_Intraparietal-3-L and S_Intraparietal-2-L<br>S_Intraparietal-2-L and S_Intraparietal-3-L<br>G_Temporal_Sup-3-L and G_Insula-posterior-1-L<br>G_Insula-posterior-1-L and G_Temporal_Sup-3-L<br>G_Temporal_Sup-4-L and G_Temporal_Sup-3-L<br>S_Sup_Temporal-3-L and G_Temporal_Sup-3-L<br>G_Temporal_Sup-3-L and G_Temporal_Sup-4-L<br>G_Temporal_Sup-3-L and S_Sup_Temporal-3-L<br>S_Sup_Temporal-4-L and S_Sup_Temporal-3-L<br>S_Sup_Temporal-5-L and S_Sup_Temporal-3-L<br>S_Sup_Temporal-3-L and S_Sup_Temporal-4-L<br>S_Sup_Temporal-5-L and S_Sup_Temporal-4-L<br>S_Sup_Temporal-3-L and S_Sup_Temporal-5-L	N_Putamen-3-L and S_Rolando-3-L<br>G_Insula-posterior-1-L and S_Rolando-4-L<br>N_Putamen-3-L and S_Rolando-4-L<br>S_Rolando-4-L and G_Insula-posterior-1-L<br>N_Putamen-3-L and G_Supp_Motor_Area-3-L<br>N_Putamen-3-L and S_Cingulate-4-L<br>N_Putamen-3-L and G_Paracentral_Lobule-2-L<br>N_Putamen-3-R and G_Paracentral_Lobule-2-R<br>N_Putamen-3-L and G_Paracentral_Lobule-3-L<br>G_Precuneus-1-R and G_Precuneus-1-L<br>G_Precuneus-1-L and G_Precuneus-1-R<br>N_Putamen-2-R and N_Putamen-2-L<br>N_Thalamus-5-R and N_Putamen-2-L<br>N_Putamen-2-L and N_Putamen-2-R<br>N_Thalamus-5-R and N_Putamen-2-R<br>S_Rolando-3-L and N_Putamen-3-L

G_Calcarine-3-R and S_Sup_Temporal-2-R<br>G_Insula-posterior-1-R and S_Sup_Temporal-3-R<br>G_Rolandic_Oper-2-R and S_Sup_Temporal-3-R<br>G_Insula-posterior-1-R and S_Sup_Temporal-4-R<br>G_Cingulum_Post-1-R and G_Cingulum_Ant-1-R<br>G_Cingulum_Post-1-R and G_Cingulum_Ant-2-R<br>G_Cingulum_Ant-1-R and G_Cingulum_Post-1-R<br>G_Cingulum_Ant-2-R and G_Cingulum_Post-1-R<br>G_Hippocampus-2-R and S_Parietooccipital-4-R<br>S_Sup_Temporal-2-R and G_Calcarine-3-R<br>S_Parietooccipital-4-R and G_Hippocampus-2-R<br>S_Postcentral-2-R and N_Putamen-3-R	S_Cingulate-3-L and G_Precuneus-3-L<br>S_Cingulate-4-L and G_Precuneus-3-L<br>S_Cingulate-5-L and G_Precuneus-3-L<br>G_Frontal_Sup-2-R and N_Putamen-2-R<br>N_Thalamus-5-R and N_Putamen-2-R<br>G_Frontal_Sup-2-R and N_Putamen-3-R<br>S_Rolando-3-R and N_Putamen-3-R<br>S_Rolando-4-R and N_Putamen-3-R<br>G_Insula-posterior-1-R and N_Putamen-3-R<br>G_Paracentral_Lobule-2-R and N_Putamen-3-R<br>S_Cingulate-4-R and N_Thalamus-5-R<br>G_Paracentral_Lobule-2-R and N_Thalamus-5-R<br>N_Putamen-2-R and N_Thalamus-5-R	G_Frontal_Inf_Tri-1-R and N_Putamen-2-R<br>G_Insula-anterior-3-R and N_Putamen-2-R<br>N_Putamen-3-R and N_Putamen-2-R<br>S_Rolando-3-L and N_Putamen-3-L<br>S_Rolando-4-L and N_Putamen-3-L<br>N_Putamen-2-R and N_Putamen-3-R<br>S_Rolando-4-L and N_Thalamus-5-L<br>G_Supp_Motor_Area-3-L and N_Thalamus-5-L<br>S_Cingulate-4-L and N_Thalamus-5-L<br>G_Paracentral_Lobule-2-L and N_Thalamus-5-L<br>N_Putamen-2-L and N_Thalamus-5-L<br>S_Rolando-4-R and N_Thalamus-5-R	S_Sup_Temporal-4-L and S_Sup_Temporal-5-L<br>G_Temporal_Mid-4-L and S_Sup_Temporal-5-L<br>G_Temporal_Inf-3-L and S_Sup_Temporal-5-L<br>S_Sup_Temporal-5-L and G_Temporal_Mid-4-L<br>S_Sup_Temporal-5-L and G_Temporal_Inf-3-L<br>N_Thalamus-5-R and G_Paracentral_Lobule-2-R<br>G_Fusiform-4-L and G_Fusiform-1-L<br>G_Fusiform-6-R and G_Fusiform-1-R<br>G_Fusiform-1-L and G_Fusiform-4-L<br>G_Fusiform-1-R and G_Fusiform-6-R<br>N_Putamen-3-R and G_Fusiform-6-R<br>G_Frontal_Sup_Orb-1-L and N_Putamen-2-L<br>G_Fusiform-6-R and N_Putamen-3-R<br>G_Paracentral_Lobule-2-R and N_Thalamus-5-R	S_Rolando-4-L and N_Putamen-3-L<br>G_Supp_Motor_Area-3-L and N_Putamen-3-L<br>S_Cingulate-4-L and N_Putamen-3-L<br>G_Paracentral_Lobule-2-L and N_Putamen-3-L<br>G_Paracentral_Lobule-3-L and N_Putamen-3-L<br>N_Thalamus-5-R and N_Putamen-3-L<br>G_Paracentral_Lobule-2-R and N_Putamen-3-R<br>N_Thalamus-5-R and N_Thalamus-5-L<br>N_Putamen-2-L and N_Thalamus-5-R<br>N_Putamen-2-R and N_Thalamus-5-R<br>N_Putamen-3-L and N_Thalamus-5-R<br>N_Thalamus-5-L and N_Thalamus-5-R

Supplementary Table 10. Component Loadings of the Structural Connectivity (DTI) Partial Least Squares Model for Language Outcomes.

**Brain Region**	**Neuroimaging Modality-Specific PLSR Models**										
	**Lesion**	**i3mT1**	**VBM_GM**	**VBM_WM**	**CBF**	**T-fMRI**	**ALF**	**FA**	**MD**	**DTI**	**rsfMRI**
	**WAB-R Spontaneous Speech**										
G_Rolandic_Oper-1-L			x							x	
G_Temporal_Sup-4-L									x	x	
N_Putamen-3-L										x	
S_Precentral-5-L				x				x			
	**WAB-R Naming**										
G_Parietal_Sup-2-L											x
N_Putamen-3-R										x	
N_Caudate-5-L											x
G_Frontal_Sup-3-L									x		x
G_Frontal_Sup-3-R			x								x
N_Putamen-3-L	x									x	
S_Intraparietal-3-R				x						x	
S_Sup_Frontal-3-L		x						x			
	**WAB-R Repetition**										
G_Frontal_Sup-2-R										x	x
G_Hippocampus-1-L		x						x			
G_ParaHippocampal-1-R		x				x					
G_Rolandic_Oper-2-R								x		x	
G_SupraMarginal-2-L				x	x						
N_Caudate-5-L			x							x	
N_Putamen-2-L			x							x	
N_Putamen-3-R										x	
S_Intraparietal-3-L				x						x	
	**WAB-R Auditory Comprehension**										
G_Temporal_Mid-1-L		x		x	x			x			
G_Angular-3-L	x		x	x							
G_Insula-anterior-2-L											x
G_Temporal_Sup-3-L				x	x	x					
N_Putamen-3-L		x								x	
G_Calcarine-1-L						x	x				
G_Calcarine-2-L							x			x	
G_Cingulum_Post-3-L		x						x			

G_Frontal_Inf_Tri-1-R										x	
G_Occipital_Mid-3-L							x				x
G_Occipital_Mid-4-L				x				x			
G_Parietal_Sup-2-L				x							x
G_Precuneus-8-R					x				x		
G_Supramarginal-4-R		x						x			
G_Temporal_Inf-1-L								x			x
G_Temporal_Inf_4-L	x		x								
G_Temp_Pole_Mid-2-L					x				x		
G_Temp_Pole_Mid-3-R										x	
N_Thalamus-3-R				x				x			
N_Thalamus-5-R										x	
S_Inf_Frontal-2-L										x	x
S_Precentral-3-R				x				x			
S_Sup_Frontal-4-R			x	x							
S_Sup_Temporal-3-L		x								x	
S_Sup_Temporal-4-l							x			x	

Supplementary Table 11. Brain Regions Uniquely Associated with WAB-R Subtest Scores Across Neuroimaging Modalities. Unique associations were determined based on averaged beta coefficients within each modality-specific PLSR model.

Supplementary Figures

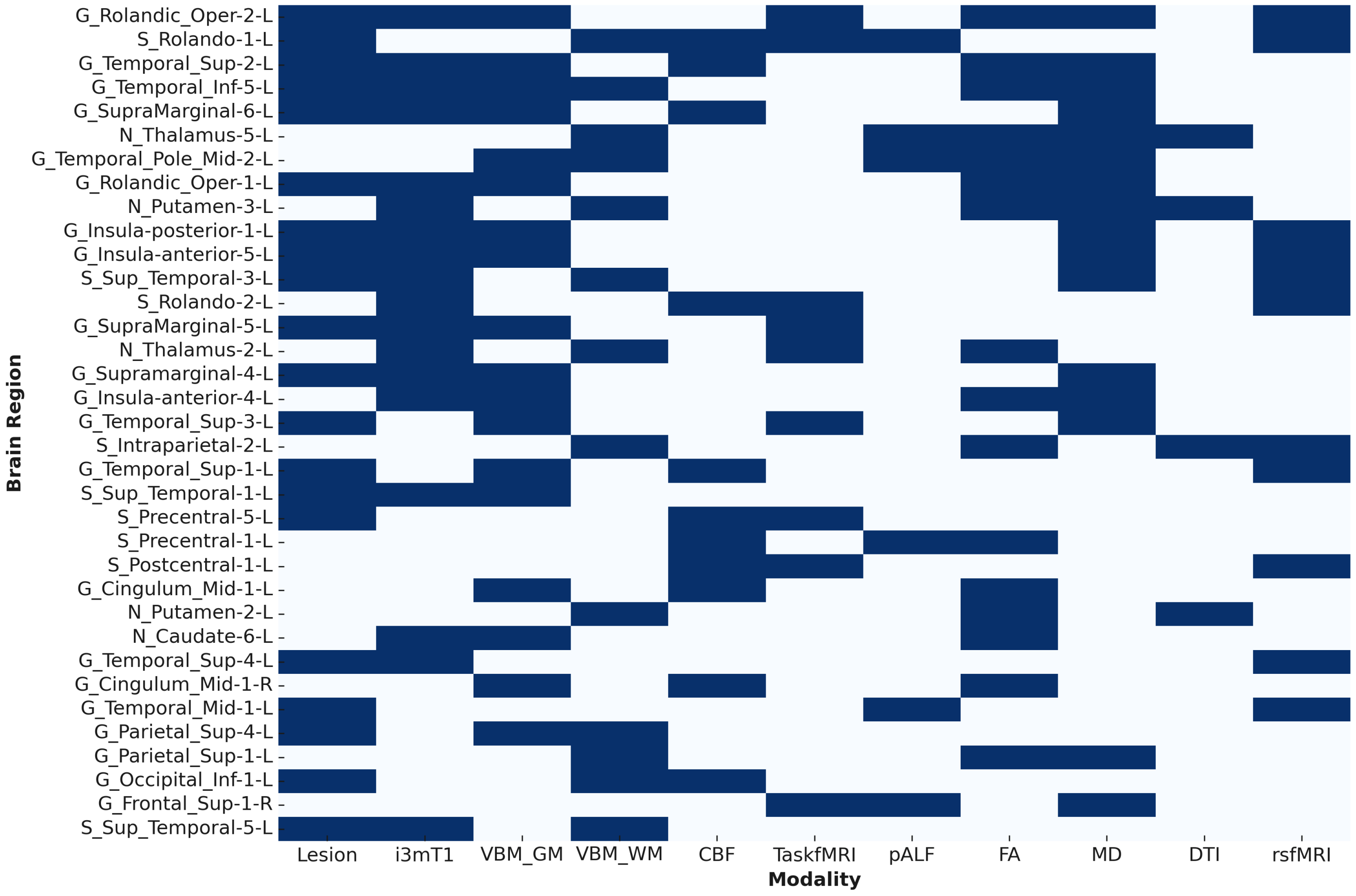

Supplementary Figure 1. Brain Regions Accounting for Shared Variance Across WAB-R Subtests. Brain regions were ranked based on VIP scores in each modality-specific PLSR model and regions involved in at least three models are visualized (overall N=86). Abbreviations: CBF=cerebral blood flow; DTI=diffusion tensor imaging; FA=fractional anisotropy; fMRI=functional magnetic resonance imaging; GM=gray matter; MD=mean diffusivity; pALF=perfusion amplitude of low-frequency fluctuations; rsfMRI=resting-state functional magnetic resonance imaging; Vbm=voxel-based morphometry; VIP=variable importance in projection; WM=white matter.

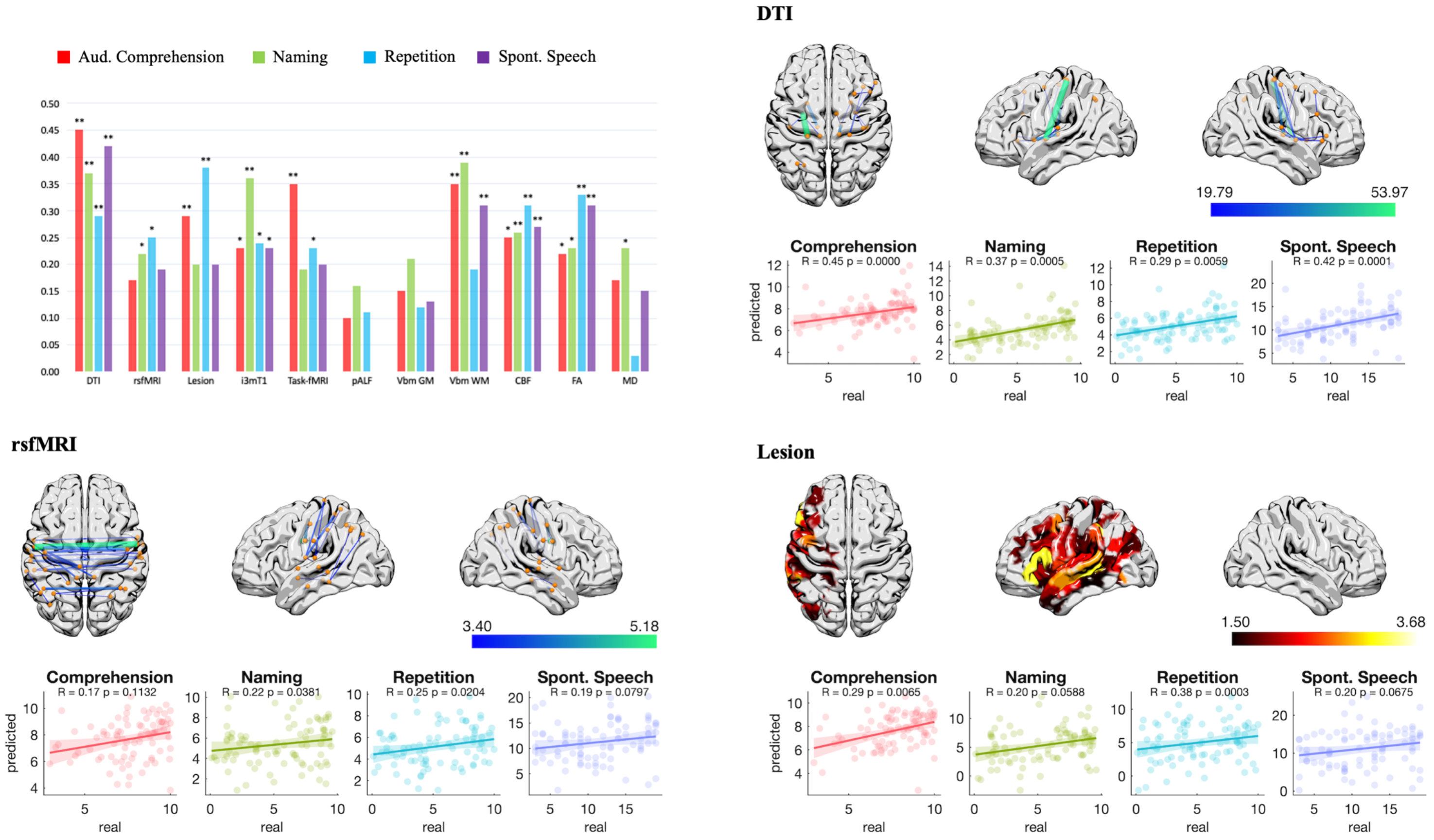

Supplementary Figure 2. Regression Estimates Relating WAB-R Subtest Scores to Modality-Specific VIP Regions (DTI, rsfMRI, Lesion). The bar plot demonstrates prediction accuracy for each WAB-R subtest based on Pearson's Correlation Coefficient (r) between actual and predicted outcomes (overall N=86). The scatter plots demonstrate the correlation between actual (X-axis) and predicted (Y-axis) and corresponding color scales reflect strength of the regression coefficient estimates. WAB-R subtest scores Abbreviations: CBF=cerebral blood flow; DTI=diffusion tensor imaging; FA=fractional anisotropy; fMRI=functional magnetic resonance imaging; GM=gray matter; MD=mean diffusivity; MNI=Montreal Neurologic Institution; pALF=perfusion amplitude of low-frequency fluctuations; rsfMRI=resting-state functional magnetic resonance imaging; Vbm=voxel-based morphometry; WAB-R=Western Aphasia Battery-Revised. WM=white matter. *p<.05; **p<.01.

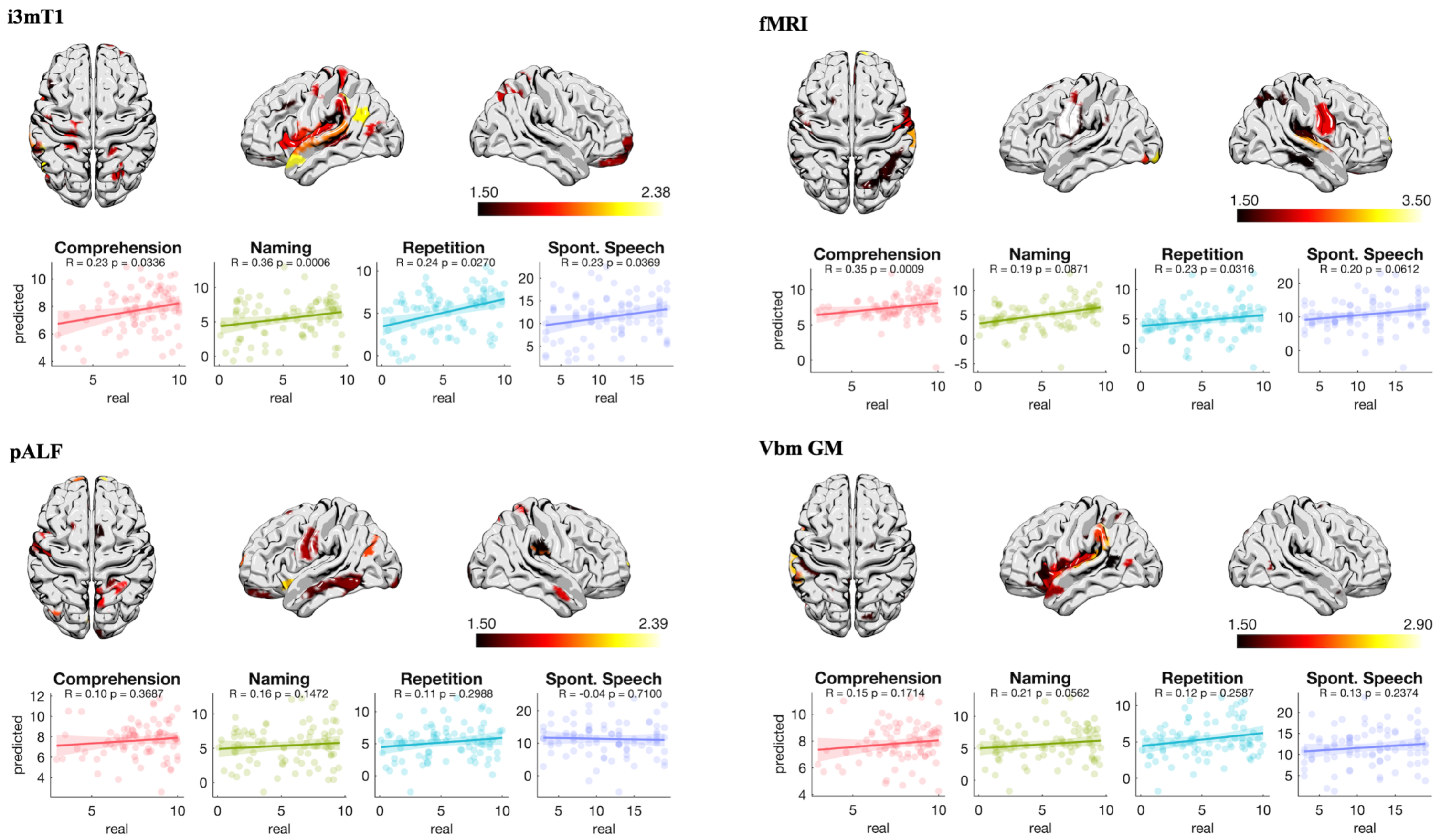

Supplementary Figure 3. Regression Estimates Relating WAB-R Subtest Scores to Modality-Specific VIP Regions (i3mT1, Task-fMRI, pALF, Vbm GM). Overall N=86. The scatter plots demonstrate the correlation between actual (X-axis) and predicted (Y-axis) and corresponding color scales reflect strength of the regression coefficient estimates. Abbreviations: fMRI=functional magnetic resonance imaging; GM=gray matter; pALF=perfusion amplitude of low-frequency fluctuations; Vbm=voxel-based morphometry. *p<.05; **p<.01.

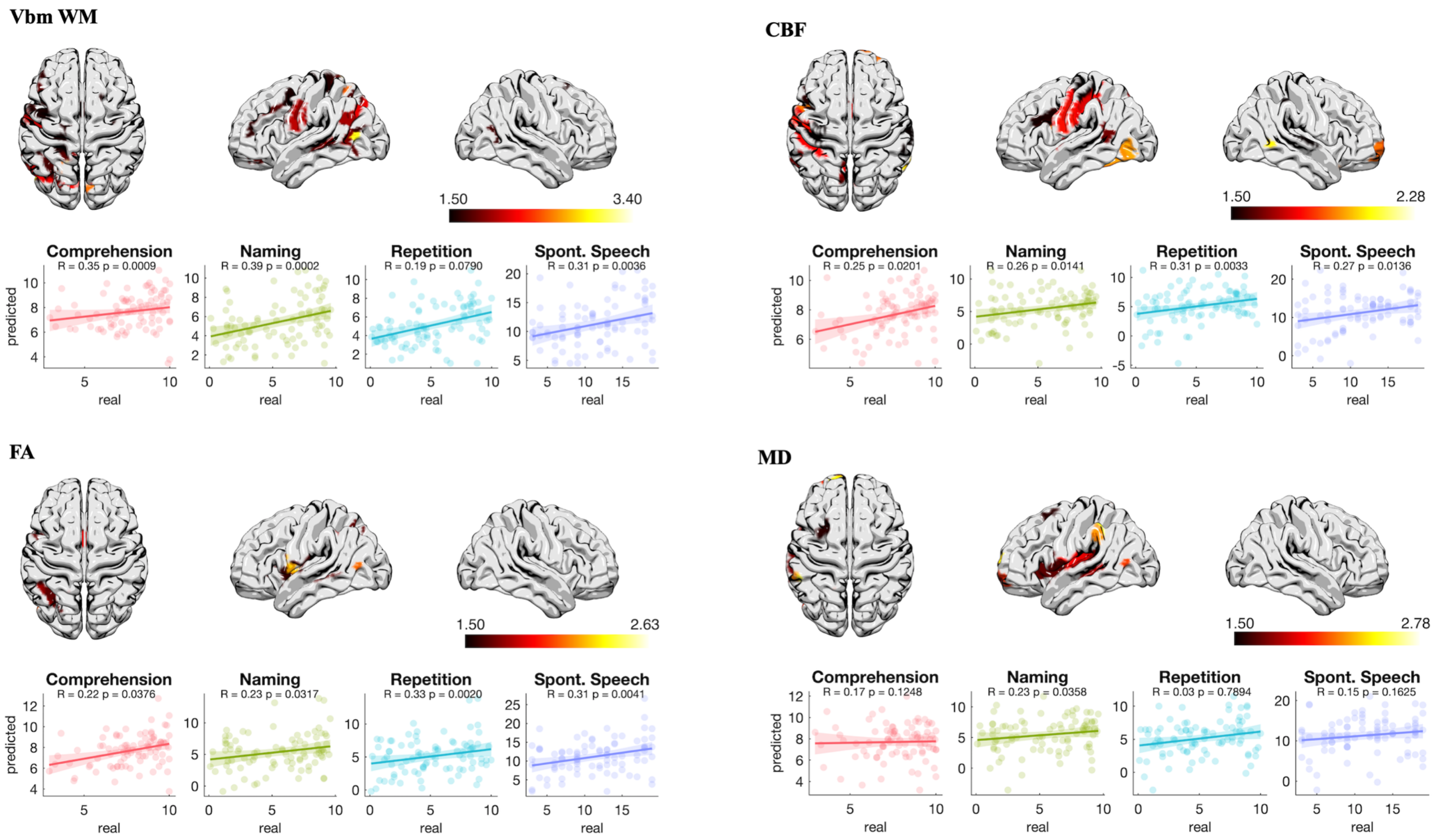

Supplementary Figure 4. Regression Estimates Relating WAB-R Subtest Scores to Modality-Specific VIP Regions (Vbm WM; CBF; FA; MD). Overall N=86. The scatter plots demonstrate the correlation between actual (X-axis) and predicted (Y-axis) and corresponding color scales reflect strength of the regression coefficient estimates. Abbreviations: CBF=cerebral blood flow; FA=fractional anisotropy; MD=mean diffusivity; Vbm=voxel-based morphometry; WM=white matter. *p<.05; **p<.01.
